# Preclinical development of a cross-protective β-SARS-CoV-2 virus-like particle vaccine adjuvanted with MF59

**DOI:** 10.1038/s41541-025-01355-y

**Published:** 2026-01-17

**Authors:** Linda Earnest, Daniel Fernandez Ruiz, Melissa A. Edeling, Julio Carrera Montoya, Ashley Huey Yiing Yap, Chinn Yi Wong, Lauren E. Holz, Stephanie Gras, Simon Collett, James P. Cooney, Kathryn C. Davidson, Samantha L. Grimley, Damian F. J. Purcell, Jason Roberts, Jamie Mumford, Chee Wah Tan, Lin-Fa Wang, Dale I. Godfrey, Matthew Frieman, Dhiraj Hans, Elizabeth Vincan, Danielle E. Anderson, Kanta Subbarao, Marc Pellegrini, Jason M. Mackenzie, Steven Rockman, William R. Heath, Joseph Torresi

**Affiliations:** 1 Department of Microbiology and Immunology, University of Melbourne, at the Peter Doherty Institute for Infection and Immunity Parkville, Melbourne, VIC Australia; 2 School of Biomedical Sciences, Faculty of Medicine & Health and the UNSW RNA Institute, The University of New South Wales, Kensington, NSW Australia; 3 Infection and Immunity Program, La Trobe Institute for Molecular Science (LIMS), La Trobe University, Bundoora, VIC Australia; 4 Department of Biochemistry and Chemistry, School of Agriculture, Biomedicine and Environment, La Trobe University, Bundoora, VIC Australia; 5 Department of Biochemistry and Molecular Biology, Monash University, Clayton, VIC Australia; 6 Department of Paediatrics, University of Melbourne Parkville, Melbourne, VIC Australia; 7 School of Science, College of Science, Engineering and Health, RMIT University, Melbourne, VIC Australia; 8 Walter and Eliza Hall Institute of Medical Research, Melbourne, VIC Australia; 9 Department of Medical Biology, University of Melbourne, Melbourne, VIC Australia; 10 Victorian Infectious Diseases Reference Laboratory, Doherty Institute for Infection and Immunity, Royal Melbourne Hospital, Melbourne, VIC Australia; 11 Programme in Emerging Infectious Diseases, Duke-NUS Medical School, Singapore, Singapore; 12 Infectious Diseases Translational Research Programme, Department of Microbiology and Immunology, Yong Loo Lin School of Medicine, National University of Singapore, Singapore, Singapore; 13 Center for Pathogen Research, Department of Microbiology and Immunology, University of Maryland School of Medicine, Baltimore, MD USA; 14 Research, Innovation & Commercialisation, Faculty of Medicine, Dentistry & Health Sciences, The University of Melbourne, Parkville, Victoria Australia; 15 Curtin Medical School, Curtin University, Perth, WA Australia; 16 WHO Collaborating Centre for Reference and Research on Influenza, VIDRL at the Doherty Institute for Infection and Immunity, Melbourne, VIC Australia; 17 Centenary Institute, The University of Sydney, Camperdown, NSW Australia; 18 Vaccine Innovation Unit, Seqirus/CSL, Parkville, VIC Australia

**Keywords:** Biotechnology, Diseases, Immunology, Microbiology

## Abstract

Whilst COVID vaccines proved to be effective in preventing severe COVID disease, they failed to control the emergence of variant viruses and antibody responses waned quickly. We report the findings of a recombinant β-SARS-CoV-2 variant virus-like particle (VLP) vaccine composed of the viral spike (S), membrane (M) and envelope (E) proteins produced in Vero cell factories. The β-SARS-CoV-2 VLP vaccine formulated with Addavax or MF59 produced strong antibody and CD4 + T cell responses and was protective in mice against pulmonary infection with Beta, Delta and Omicron BA.5 variant viruses. Multiplex RBD-ACE2 binding inhibition assay was performed as a surrogate virus neutralisation test and revealed immune sera from immunised mice produced low-titre broad-inhibitory anti-RBD-ACE2 antibodies (sNAb) to Alpha, Delta, Beta, Gamma, Mu, Omicron BA.1, BA.2, BA.5 and XBB1.5. However, microneutralisation assays did not show the presence of sNAb. The β-SARS-CoV-2 VLP is strongly immunogenic producing broad antibody and T cell responses and is protective against infection with SARS-CoV-2 variant viruses.

## Introduction

COVID-19 has presented the greatest global health challenge in over 100 years with over 700 million infections and more than 7 million deaths recorded before the pandemic was declared over in May of 2023. Globally, we experienced repeated waves of infection fuelled by the relentless emergence of new variants that became better adapted at infecting as well as escaping vaccine, therapeutic monoclonal antibodies and infection induced immunity whilst still causing significant morbidity and mortality^1–5^. Although current COVID-19 vaccines have proven to be highly effective at preventing severe disease^6,7^, their efficacy has waned against the Omicron variants highlighted most significantly by Omicron subvariants BQ.1.1, BA2.75.2, XBB.1.5, BA2.86, EG.5, JN.1 and KP1-3^8–11^. Evidence to date suggests that bivalent vaccines containing the spike of Omicron BA.5 and XBB.1.5 provide potent neutralising antibody (NAb), and broad cross reactivity against Omicron variants including BQ.1.1, BA.2.25, BA.4.6, BF.7 and XBB.1 but have become less effective against emerging variants like Omicron LF.7 and NB.1.8.1^11–18^. The rapid waning of vaccine immunity will favour the spread of variants and may also contribute to selecting out vaccine escape variants as NAb titres wane to sub-neutralising levels^19^.

The importance of pursuing more effective vaccines to combat SARS-CoV-2 variants is also highlighted by studies showing that vaccination has a significant impact on reducing progression to severe COVID-19 and death^20–22^. Vaccination remains an important strategy to maintain as infection alone in the absence of vaccination reportedly provides limited cross-protective immunity against Omicron^23^.

We have developed a unique, adaptable SARS-CoV-2 VLP vaccine platform that enables us to produce VLPs from a single self-cleaving polyprotein containing the viral structural proteins of interest^24^. Our SARS-CoV-2 VLP vaccine includes the key structural proteins of SARS-CoV-2, spike (S), envelope (E) and membrane (M), which have been shown in several studies to be important in producing protective and memory CD4+ and CD8 + T cell responses^25–29^. Both E and M proteins are also highly conserved across SARS-CoV-2 viruses. Our vaccine offers some advantages over other approaches for the development of viral vaccines including: (i) the delivery of multiple viral proteins in a particulate structure, unlike current monovalent and bivalent mRNA COVID-19 vaccines that only deliver the viral spike (S) protein gene (ii) avoidance of adverse events associated with mRNA vaccines (eg, severe allergic reactions and myocarditis) that have also contributed to vaccine hesitancy (iii) its independence of mRNA, thereby avoiding limitations on dosing due to mRNA’s reactogenicity and removing constrains on the development of polyvalent vaccines and (iv) potentially delivering long lasting protective immune responses to overcome the need for frequent boosting required with mRNA vaccines.

In this study, we report the development of a unique ß-variant SARS-CoV-2 VLP vaccine. The value in this approach is underpinned by several reports showing that ß-vaccines induce broad cross-protection against several variants, including Alpha, Beta, Delta, Gamma, Mu and Omicron subvariants^16,30–34^. Our vaccine also contains the N-terminal domain (NTD) and S2 of ß-SARS-CoV-2 S protein, regions that may contribute to producing important cross-protective antibody responses^35,36^.

We can scale up the production of our ß-SEM-SARS-CoV-2 VLP vaccine using methods compatible with industry standards. By formulating our ß-SEM-SARS-CoV-2 VLPs with MF59 the vaccine is strongly immunogenic with enhanced antibody and T cell responses and is completely protective against in vivo challenge with SARS-CoV-2 variants in a mouse model. Finally, the inclusion of an interchangeable S protein allows us to introduce variant S protein sequences reflective of emerging SARS-CoV-2 viruses. The results from this adaptable vaccine platform provides proof of concept for the development of effective next-generation VLP COVID vaccines.

## Results

### Production of recombinant rAd-S-SARS-CoV-2 and rAd-E_SPP_M-SARS-CoV-2 and infection of Vero cells

We have previously described the production of SARS-CoV-2 VLPs using a recombinant adenovirus vector carrying a gene construct containing the full-length S separated from the E gene by an SP/SPP sequence and the same sequence between the E and M genes^24,37^. To produce a vaccine with broader immunogenicity, we exchanged the Ancestral S gene with that of Beta SARS-CoV-2. The Beta spike protein was stabilised by the introduction of two proline substitutions A892P and A942P^38^.

**Fig. 1:**
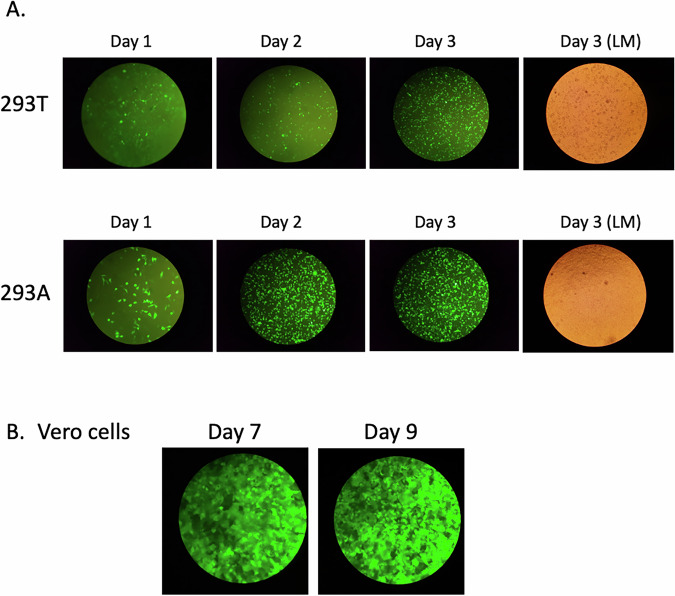
Transduction efficiency of 293T versus 293A HEK cells. **A** Transduction of 293A and 293T HEK cells with rAd-ß-S or rAd-E_SPP_M virus. Cells were infected in 6-well plates at an MOI of 1. An approximate number of GFP-positive 293A HEK cells was determined after 24, 48 and 72 h by fluorescence microscopy for GFP expression. The number of GFP-positive 293A HEK cells (lower panel) was substantially greater than 293T HEK cells (upper panel), with ~80% of 293A HEK cells positive as early as day 2. **B** Transduction of Vero cells with rAd-ß-S plus rAd-E_SPP_M virus. Cells were infected in 6-well plates at an MOI of 1 for both viruses. This resulted in almost 100% transduction of cells by day 7 pi.

These adenoviruses (containing Beta S protein and Ancestral E and M proteins) were used to transduce 293T or 293A HEK cells to determine which cell line resulted in the greatest transduction efficiency. Infection of 293A HEK cells produced a larger number of infected cells over a 3-day time course than 293T cells, confirming a higher transduction efficiency (Fig. 1A). Consequently, adenoviruses were amplified by passaging in 293A HEK cells and the rAd-ß-S-SARS-CoV-2 and rAd-E_SPP_M-SARS-CoV-2 viruses produced were used to co-transduce Vero cells at an MOI of 1. By day 7, virtually 100% of Vero cells were positive, as determined by the expression of a GFP reporter (Fig. 1B).

### Production and characterisation of ß-SARS-CoV-2 VLPs

We previously demonstrated production of SARS-CoV-2 VLPs from a single^24,37^ or three separate adenoviruses expressing the S, E and M genes of SARS-CoV-2^39^. We now wanted to determine whether producing VLPs could be improved by infecting Vero cells with two separate recombinant adenoviruses, one carrying the Beta-S gene and the second, the EM genes separated by the SP/SPP sequence. This approach would also provide the capacity to more readily alter the S protein to reflect current circulating viral variants.

Vero cell factories were transduced with rAd-ß-SE_SPP_M-SARS-CoV-2 or rAd-ß-S-SARS-CoV-2 plus rAd-E_SPP_M-SARS-CoV-2 and VLPs were purified from culture supernatants. The yield of purified VLPs was more than 2-fold higher in cultures transduced with rAd-ß-S-SARS-CoV-2 plus rAd-E_SPP_M-SARS-CoV-2 (average 3.7 mg) compared with rAd-ß-SE_SPP_M-SARS-CoV-2 virus (average 1.5 mg).

**Fig. 2:**
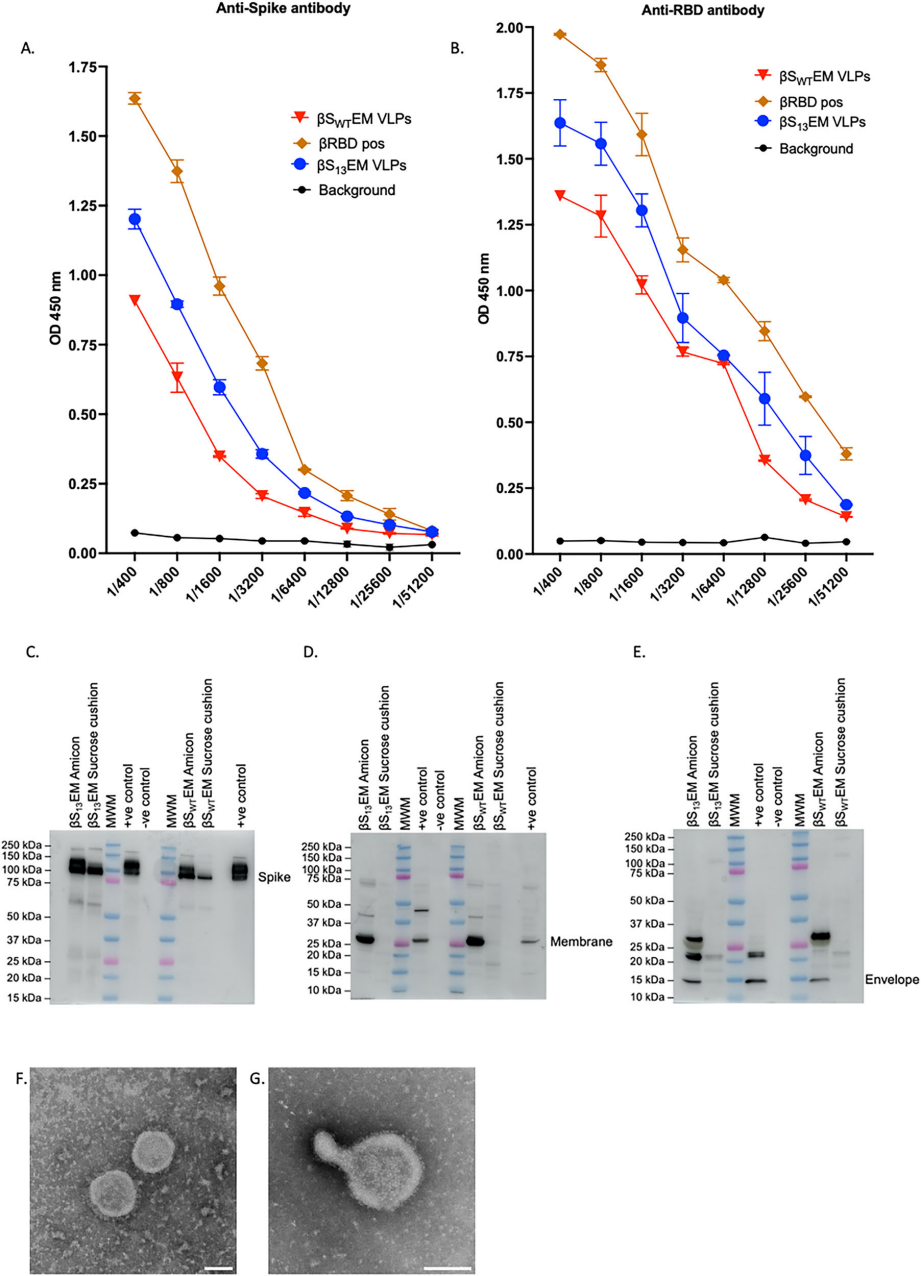
Characterisation of ß-S_13_EM-SARS-CoV-2 VLPs. ELISA analysis of ß-S_13_EM-SARS-CoV-2 VLPs and ß-S_WT_EM-CoV-2 VLPs for spike (**A**) and RBD (**B**) proteins. Plates were coated with ß-S_WT_EM-CoV-2 VLPs or ß-SARS-CoV-2 RBD followed by probing with either anti-Spike or anti-RBD antibody in 2-fold dilutions from 1:400 to 1:51200 (mean ± SD). ELISAs were performed three times and each dilution tested in triplicate. Western immunoblot analysis of ß-S_13_EM-SARS-CoV-2 VLPs compared with ß-S_WT_EM-CoV-2 VLPs for the presence of (**C**) Spike protein (**D**) Membrane and (**E**) Envelope protein. Amicon ultrafiltration consistently resulted in a higher yield S, M and E proteins than sucrose cushion ultracentrifugation as shown by Western blot analysis of the VLPs (**F**) Negative stain electron microscopy showing morphology of (**F**) ß-S_13_EM-SARS-CoV-2 VLPs and (**G**) ß-S_WT_EM-CoV-2 VLPs.

During the cloning of the ß-S gene into the adenoviral vectors, one clone (S_13_) was identified that included a 22 amino acid extension at the C-terminal end of the S protein (rAd-ß-S_13_)^40^. The two ß-S containing VLPs were compared in a small-scale preparation and analysed as before by ELISA for ß-S protein and ß-RBD (Fig. 2A, B), Western immunoblots for S, M and E proteins (Fig. 2C–E) and electron microscopy (Fig. 2F, G). Interestingly, ß-S_13_EM-SARS-CoV-2 VLPs produced stronger reactivity by ELISA for both S and ß-RBD than ß-S_WT_EM-CoV-2 VLPs (Fig. 2A, B).

**Fig. 3:**
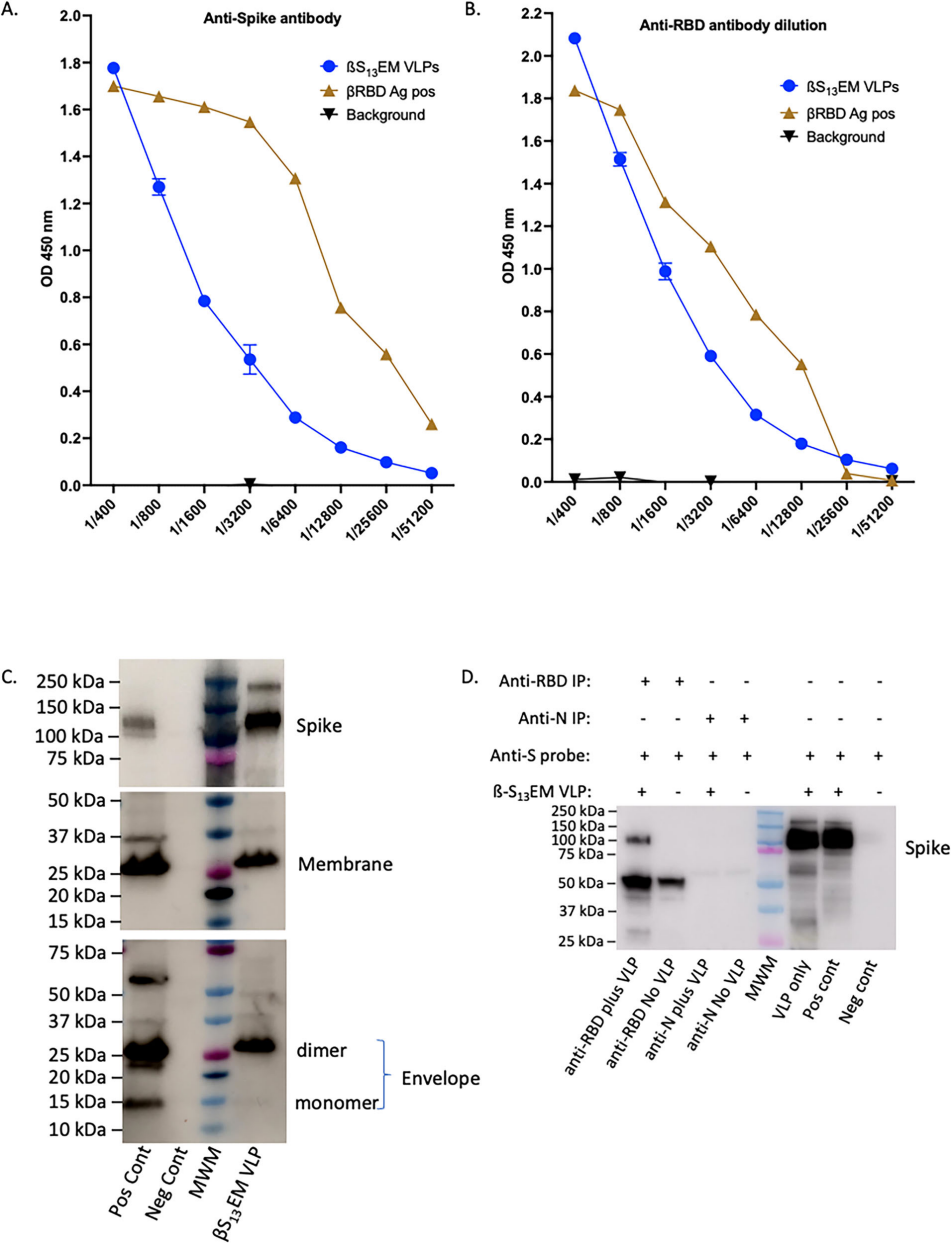
Characterisation of ß-S_13_EM-SARS-CoV-2 VLPs from large scale production. ELISA analysis of ß-S_13_EM-SARS-CoV-2 VLPs for spike (**A**) and RBD (**B**) proteins. Plates were coated with ß-S13EM-SARS-CoV-2 VLPs followed by probing with either anti Spike or anti-RBD antibody in 2-fold dilutions from 1:400 to 1:51200 (mean ± SD). ELISAs were repeated three times and each dilution tested in triplicate (**C**) Western immunoblot analysis of ß-S_13_EM-SARS-CoV-2 VLPs showing the presence of spike, membrane and the dimeric form of envelope protein. **D** Immunoprecipitation of ß-S_13_EM-SARSCoV-2 VLPs with anti-Wu-RBD antibody and probing with anti-Wu-S antibody. Anti-N antibody failed to immunoprecipitate VLPs. ß-S_13_EM-SARS-CoV-2 and Ancestral SARS-CoV-2 VLPs were included as positive controls for S protein.

To test the yield from a large-scale production, Vero cell factories were transduced with rAd-ß-S_13_ plus rAd-E_SPP_M and VLPs purified from culture supernatants. The yields of purified ß-S_13_EM-SARS-CoV-2 VLPs were consistently higher than the single wild-type type rAd-ß-SE_SPP_M (8-fold; average 12.2 mg vs 1.5 mg per 500 ml cell culture supernatant) or the dual rAd-ß-S plus rAd-E_SPP_M (3-fold; average 12.2 mg vs 3.7 mg per 500 ml cell culture supernatant) (Table 1). The ß-S_13_EM-SARS-CoV-2 VLPs were characterised by ELISA for ß-S protein and ß-RBD (Fig. 3A, B) and Western immunoblots for S, M and E proteins (Fig. 3C). Consistent with a recent report characterising the oligomeric forms of E in SARS-CoV-2 virus, the E protein in VLPs was present as a dimer (Fig. 3C, lower panel)^41^ in contrast to earlier reports suggesting that E forms a pentameric structure^42^. Furthermore, studies have shown that resolution of the E protein by SDS-PAGE reveals the formation of dimers that are stabilised by leucine-valine side chain interactions between the transmembrane sequences of opposing E proteins that are almost analogous to a leucine zipper^43^.

**Table 1:** Yield of ß-SEM-SARS-CoV-2 VLPs according to rAdenoviruses used to transduce cell factory cultures

	rAd-ß-SE_SPP_M	rAd-ß-S plus rAd-E_SPP_M	rAd-ß-S_13_ plus rAd-E_SPP_M
Yield (mg/500 ml)	1.5	3.7	12.2

To further analyse the S protein contained in the VLPs, we immunoprecipitated ß-S_13_EM-SARS-CoV-2 VLPs with anti-Wu-RBD antibody and probed Western blots using anti-Wu-S-antibody. The VLPs were immunoprecipitated with anti-Wu-RBD antibody but not with anti-N antibody (negative control), confirming that the S protein contained RBD (Fig. 3D). The purified ß-S_13_EM-SARS-CoV-2 VLP preparation was also tested for endotoxin and found to have very low levels of 3.8 EU/mL (0.07 EU/20 µg dose) of endotoxin.

**Fig. 4:**
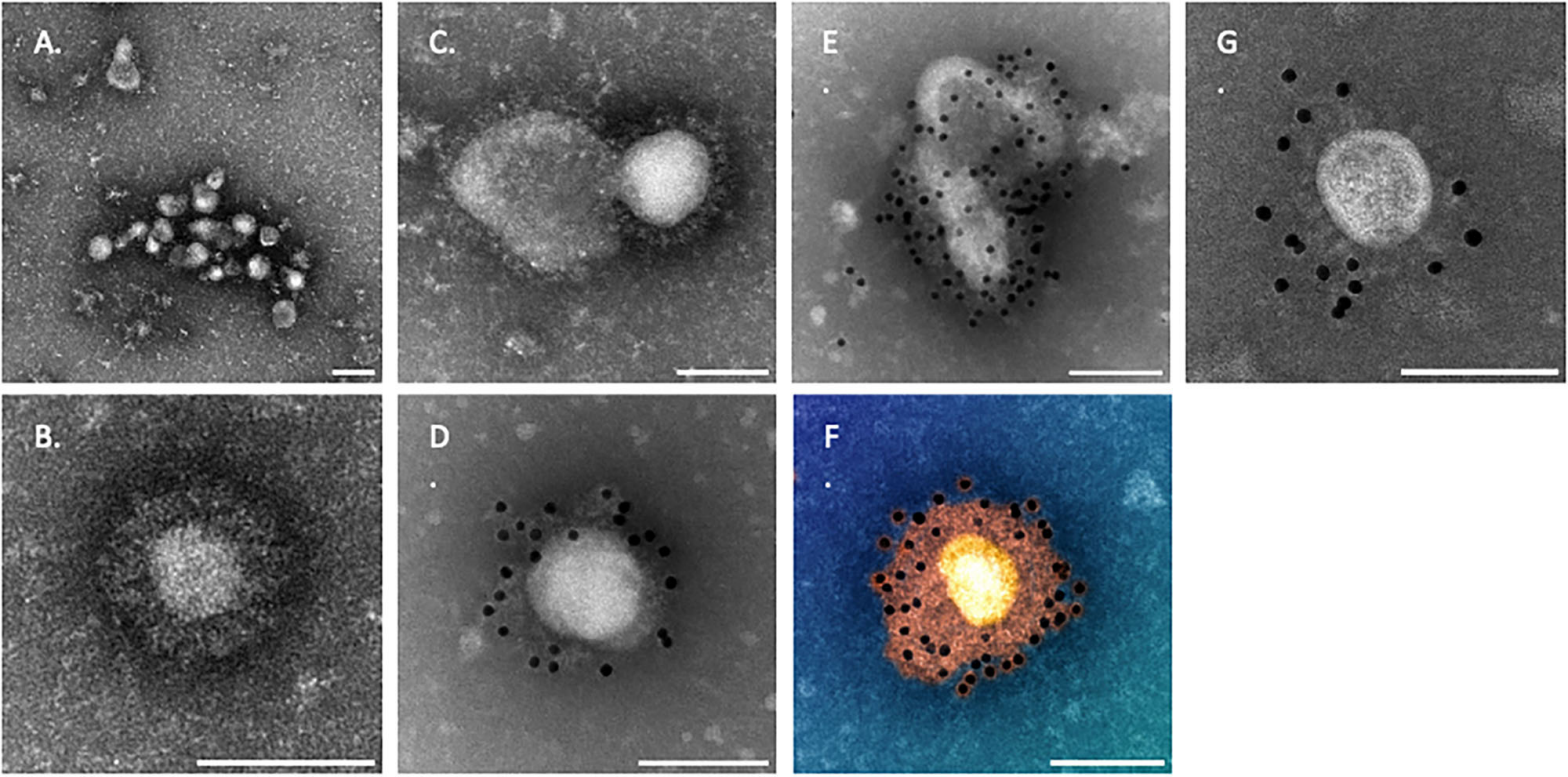
Electron microscopy of ß-S_13_EM-SARS-CoV-2. **A**, **B**, **C** Transmission EMs showing ß-S_13_EM-SARS-CoV-2 VLPs of 50-100 nm and with characteristic spikes. **D**, **E** Immunogold EM showing binding of gold beads with anti-ß-RBD antibody to RBDs of spikes on a single VLP and cluster of VLPs. **F** Colour enhanced image of Immunogold EM highlighting the spacing of anti-ß-RBD coated gold beads to spikes of a ß-S_13_EM-SARS-CoV-2 VLP. **G** IEM of Delta SARS-CoV-2 virus as a positive control. Scale bar = 100 nm.

To confirm that the S, M and E proteins assembled into VLPs, we performed negative stain electron microscopy (EM) (Fig. 4A, B, C) and immunogold EM with anti-ß-RBD antibody (Fig. 4D, E, F, respectively). ß-S_13_EM-SARS-CoV-2 VLPs consisted of pleomorphic 50 to 100 nm particles richly decorated with characteristic spikes. Immunogold EM demonstrated the binding of gold beads to RBDs of spikes (Fig. 4D, E, F) and this was comparable to the binding to RBDs of spikes of Delta SARS-CoV-2 virus (Fig. 4G).

### In vivo antibody responses to ß-S_13_EM-SARS-CoV-2/Addavax VLPs

**Fig. 5:**
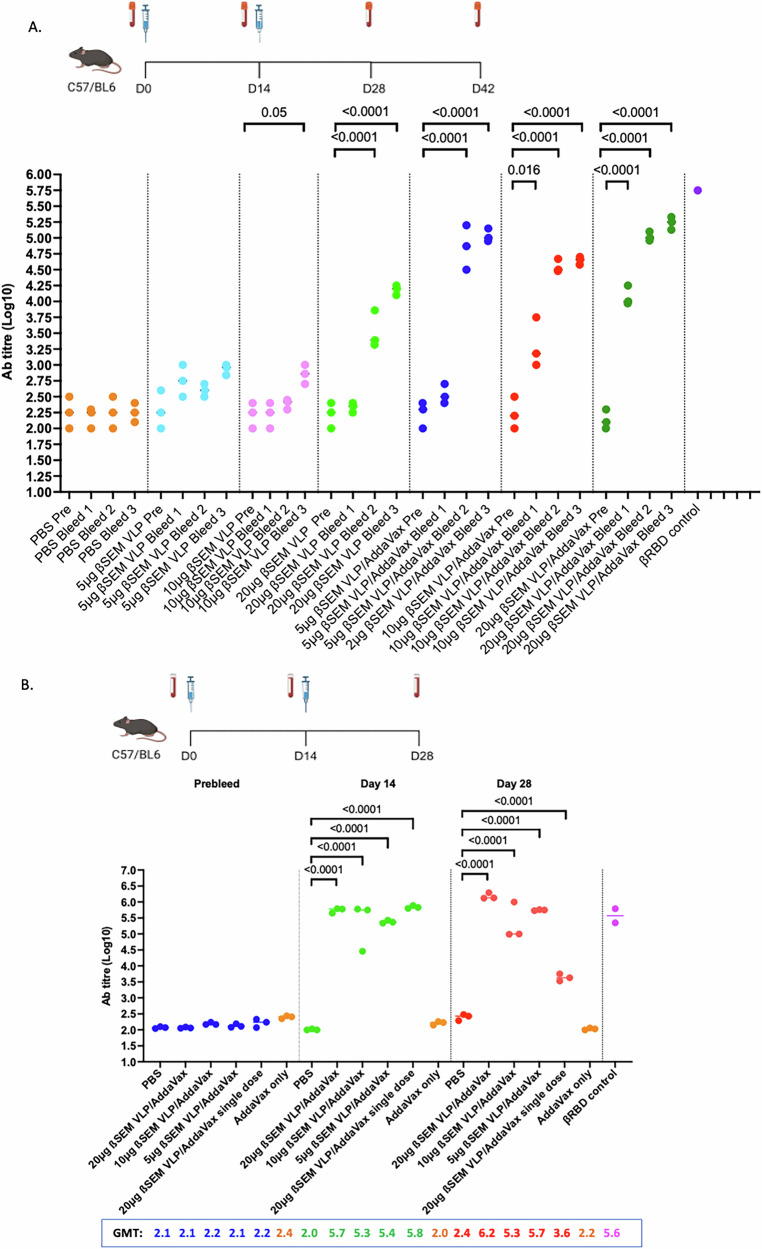
Antibody responses to ß-S_13_EM-SARS-CoV-2 VLP/Addavax vaccine. **A** Mice were immunised with 2 doses of 5, 10 or 20 µg of vaccine with or without adjuvant 2 weeks apart, bled on days 0,14, 28 and 42. Anti-ß-RBD antibody responses were assessed using ELISA, with durable responses detected up to day 42. Immunisations were performed twice with 5 mice in each vaccine group. Sera for each group at respective time points were pooled and tested in triplicate. ELISAs were performed twice. Data from groups of mice were compared using One-Way ANOVA and Tukey’s Multiple Comparisons test. Bar indicates mean value; each dot represents one mouse. **B** Mice were immunised with 2 doses of 5, 10 or 20 µg of vaccine with Addavax 2 weeks apart, bled on days 0,14 and 28. Anti-ß-RBD antibody responses were assessed using ELISA, as before. Immunisations were performed twice with 5 mice in each vaccine group. Sera for each group at respective time points were pooled and tested in triplicate. ELISAs were performed twice. Data from groups of mice were compared using One-Way ANOVA and Tukey’s Multiple Comparisons test. Geometric mean titre (GMT) values are displayed for each group at day 0,14 and 28. (Immunisation schedule Figs. 5A, B were created with BioRender.com).

We next determined the immunogenicity of our ß-S_13_EM-SARS-CoV-2 VLP vaccine in mice. Mice were vaccinated twice, 2 weeks apart, with 5, 10 or 20 µg of ß-S_13_EM-SARS-CoV-2 VLPs alone or mixed with Addavax. In the absence of adjuvant, both 5 and 10 µg doses resulted low-titre antibody responses. A 20 µg dose produced modest antibody responses with titres of 3.5 log_10_ after 28 days and 4.25 log_10_ at day 42 (Fig. 5A). In contrast, vaccine mixed with Addavax produced strong antibody responses in all dose groups. Antibody titres peaked after day 28 and were maintained to day 42 (Fig. 5A).

Adjuvanted vaccine produced stronger sustained antibody responses peaking by day 28 compared to non-adjuvanted vaccine. We next determined whether a single dose of 20 µg of ß-S_13_EM-SARS-CoV-2 VLPs with Addavax could produce similar antibody responses to two doses of 5, 10 or 20 µg vaccine 14 days apart. Mice in all groups developed strong antibody responses as early as day 14, with titres between 5 and 6 Log_10_ (Fig. 5B). Antibody responses were maintained at day 28 in mice given two doses of vaccine. In contrast, antibody titres waned by day 28 in mice receiving only a single dose of vaccine (Fig. 5B), highlighting the importance of administering two doses of vaccine.

**Fig. 6:**
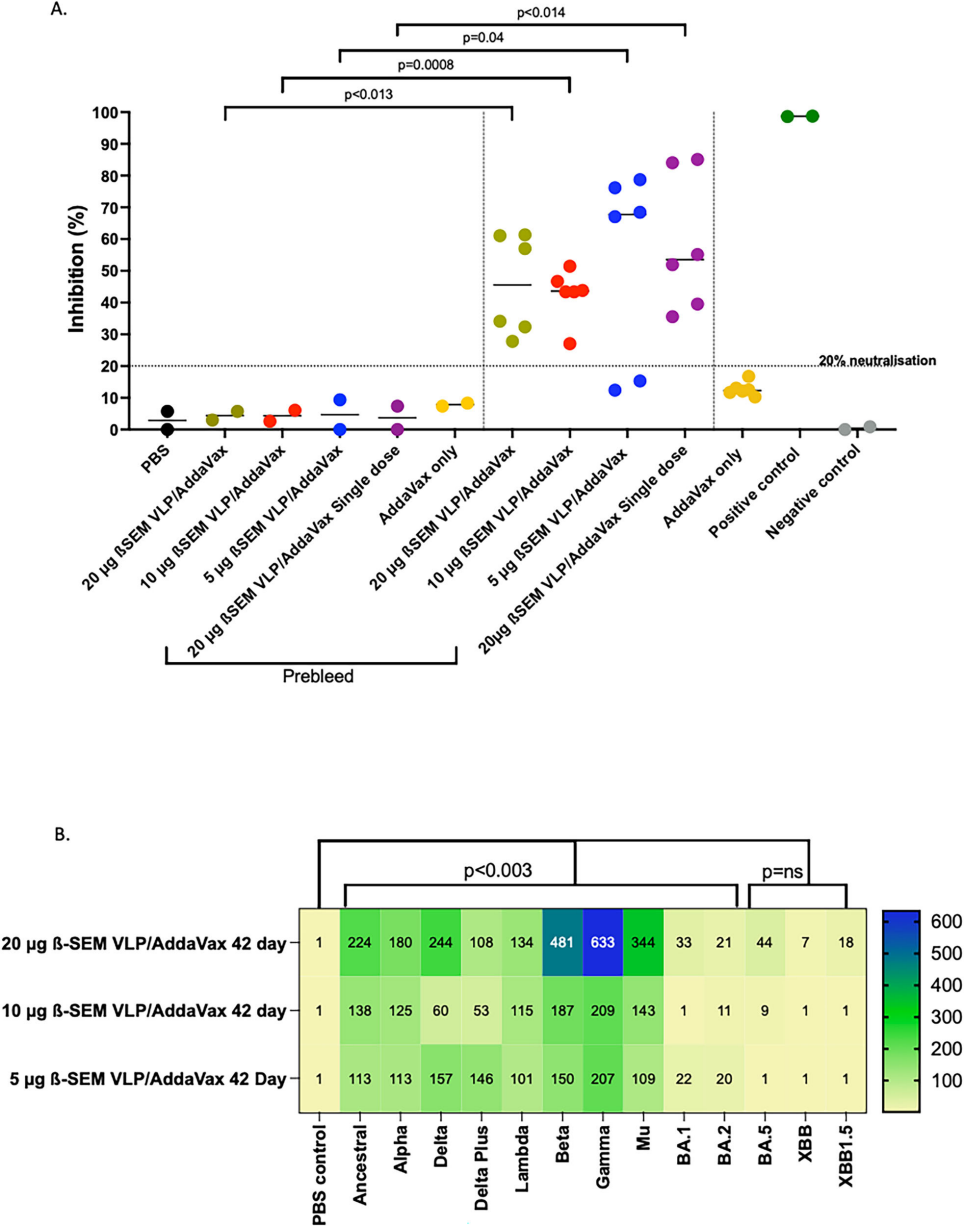
Breadth of antibody responses to ß-S_13_EM-SARS-CoV-2 VLP/Addavax vaccine. **A** sVNT assay (with Ancestral RBD-HRP) was performed on immune sera from mice immunised with ß-SARS-CoV-2 VLPs formulated with Addavax, or PBS only and compared to respective prebleed samples. For prebleed samples, owing to small volumes collected, sera from respective groups were pooled and tested in duplicate. Day 28 sera for each mouse in each vaccine group were tested individually in duplicate. Data were analysed using one-way ANOVA and Tukey’s Multiple Comparisons test. **B** Samples from day 42 were tested in a multiplex sVNT inhibition assay for their ability to inhibit the binding to ACE2 to SARS-CoV-2 Alpha, Beta, Gamma, Delta, Delta + , Lambda, Mu, Omicron BA.1, BA.2, BA.5, XBB.1 and XBB.1.5. Multiplex assays with sera from two separate immunisation series were performed in duplicate. The arithmetic mean titres of the half maximal inhibitory dilution (ID_50_) are shown for each sample and the range of titres shown in the legend bar.

We then used a surrogate virus neutralisation test (sVNT) to characterise antibody responses in sera collected on day 28 from immunised mice. This assay assesses antibodies that inhibit the binding of SARS-CoV-2 RBD to human ACE2 and provides a surrogate measure of neutralising antibodies (sNAb)^44^. Sera from immunised mice inhibited binding of SARS-CoV-2 RBD to human ACE2 by 30 to 85% (Fig. 6A). Two mice immunised with 5 µg of with ß-S_13_EM-SARS-CoV-2 VLP/Addavax failed to develop detectable sNAb responses in this assay. In contrast, mice immunised with a single dose of 20 µg of ß-S_13_EM-SARS-CoV-2 VLP/Addavax developed sNAb responses (Fig. 6A).

We also examined the breadth of sNAb responses in mice using a multiplex RBD-ACE2 binding inhibition assay (Fig. 6B)^45^. Neutralising activity (indicated as half-maximal inhibitory dilution, ID50) was observed in end-point sera from all groups of mice against Alpha, Delta, Lambda, Beta, Gamma and Mu, albeit at low titre. The anti-RBD activity was greater in the day 42 sera for the 20 µg/Addavax group compared to the 5 and 10 µg/Addavax groups (Fig. 6B). The activity against Omicron BA.1, BA.2, BA.5, XBB and XBB.1.5 was negligible in all groups (Fig. 6B).

As a final approach to test for the presence of sNAb, we performed microneutralisation assays. Two groups of 5 C57BL/6 mice were immunised with two doses of 10 µg of ß-SARS-CoV-2 VLP vaccine adjuvanted with Addavax, 14 days apart. Mice were bled on days 0, 14 and euthanised on day 28. Sera from day 28 were tested in microneutralisation assays against Ancestral (Wuh1), Beta (B.1.351) and Omicron BA.5 SARS-CoV-2 viruses. The immune sera did not appreciably neutralise viral entry of the Ancestral, Beta or Omicron BA.5 viruses (Supplementary Fig. 1).

### ß-S_13_EM-SARS-CoV-2 VLPs produce CD4+ and CD8 + T cell responses

**Fig. 7:**
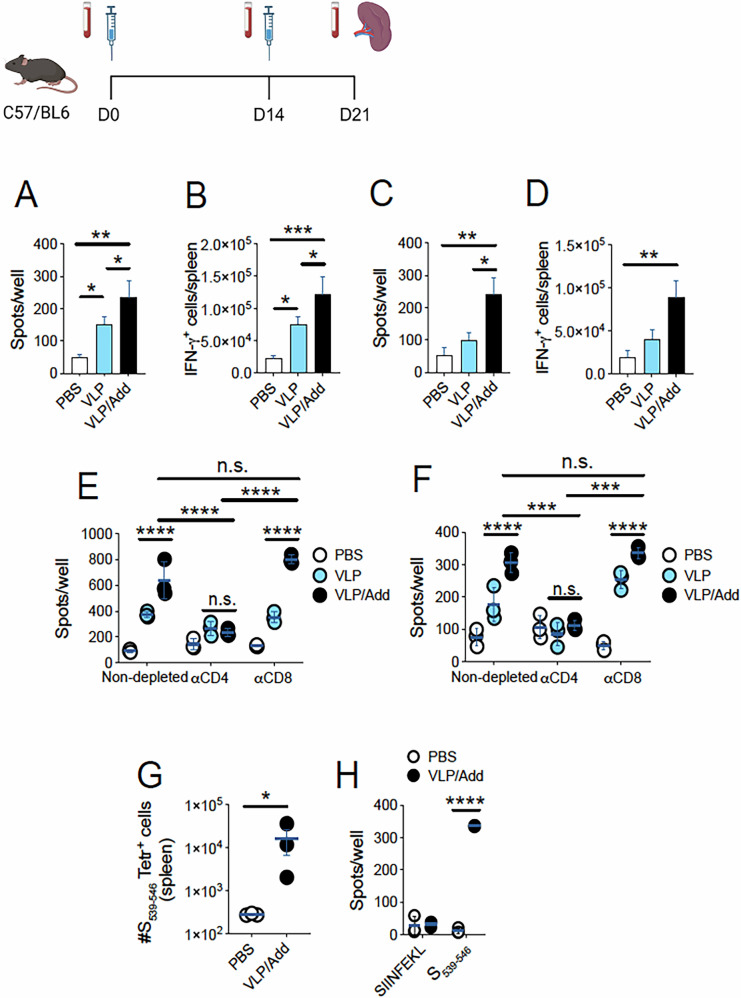
Detection of T cell responses elicited by the ß-S_13_EM-SARS-CoV-2 vaccine formulated with Addavax. **A**, **B** C57BL/6 mice were injected with two doses of 10ug ß-S_13_EM-SARS-CoV-2 VLP, i.m., formulated or not with Addavax (50% vol/vol) 14 days apart. Control mice received PBS. Mice were euthanised on day 21 and splenocytes (2 × 10^5^ per well) were restimulated in vitro using primary DC from naïve mice (2 × 10^5^ per well) pre-cultured with 5 µg/well ß-S_13_EM-SARS-CoV-2 VLP. Numbers of IFN-γ-producing cells were measured using ELISpot. All spleens in each group (*n* = 3) were pooled and analysed in triplicate wells per group. Data are from a single experiment and were analysed using one-way ANOVA. **A** Number of spots/well. **B** Average total number of responding splenocytes per mouse. Data were log-transformed and compared using One-Way ANOVA and Tukey’s Multiple Comparisons test. Data are from a single experiment with 3 mice/group, and were log-transformed and compared using one-way ANOVA and Tukey’s Multiple Comparisons test. **C**, **D** As in (**A**, **B**), but mice were vaccinated with 20 µg ß-S_13_EM-SARS-CoV-2 VLPs. In this case, 10^5^ primary DC were used per well for in vitro restimulation. Data are from a single experiment with three mice/group, and were log-transformed and compared using one-way ANOVA and Tukey’s Multiple Comparisons test. **E** Mice were vaccinated and splenocytes extracted, restimulated with ß-S_13_EM-SARS-CoV-2 VLPs in vitro, and screened for IFNγ production using ELISpot as in **A** (10 µg ß-S_13_EM-SARS-CoV-2 VLP dose), but CD4 or CD8 T cells were depleted prior to culture using magnetic beads mixed with anti-CD4 and anti-CD8 antibodies. Non-depleted samples were incubated with beads without antibodies. Data are from a single experiment and were log-transformed and analysed using two-way ANOVA and Tukey’s multiple comparisons test to account for comparisons between the variables of immunisation groups and treatment groups. **F** Mice were vaccinated and splenocytes extracted, restimulated with ß-S_13_EM-SARS-CoV-2 VLPs in vitro, and screened for IFNγ production using ELISpot as in **C** (20 µg ß-S_13_EM-SARS-CoV-2 VLP dose), but CD4+ or CD8 + T cells were depleted prior to culture using magnetic beads mixed with anti-CD4 and anti-CD8 antibodies. Non-depleted samples were incubated with beads without antibodies. Data are from a single experiment and were log-transformed and analysed using two-way ANOVA and Tukey’s multiple comparisons test. **G** Detection of CD8 + T cell responses elicited by the ß-S_13_EM-SARS-CoV-2 vaccine formulated with Addavax. C57BL/6 mice were vaccinated twice with 20 µg ß-S_13_EM-SARS-CoV-2 VLPs as in C. S_539-546_ Tetramer staining was used to identify Spike-specific CD8 + T cells among splenocytes. Data are representative of three independent experiments and were log transformed and compared using an unpaired t-test. **H** Mice were vaccinated twice with 20 µg of ß-S_13_EM-SARS-CoV-2 VLPs as in C, and CD8 T cells were enriched from their spleens, and restimulated in vitro using S_539-546_ peptide. ELISpot was performed to detect IFNγ responses. Data are from a single experiment and were log-transformed and analysed using two-way ANOVA and uncorrected Fisher’s Least Significant Difference test (Immunisation schedule figure created with BioRender.com).

We sought to determine whether our ß-S_13_EM-SARS-CoV-2 VLP vaccine elicited measurable T cell responses in mice. Mice were vaccinated twice, 2 weeks apart, with 10 µg (Fig. 7A, B, E) or 20 µg ß-S_13_EM-SARS-CoV-2 VLPs (Fig. 7C, D, F) per dose, adjuvanted with Addavax or not, and euthanised one week after the last vaccine dose. Splenocytes were then assessed for their capacity to respond to VLPs using ELISpot. A clear response was detected by splenocytes from VLP/Addavax vaccinated mice in contrast to unvaccinated mice (Fig. 7A-D). Mice vaccinated with ß-S_13_EM-SARS-CoV-2 VLP without adjuvant also developed a detectable response above background but of less intensity than those in the VLP/Addavax group (Fig. 7A-D). Indeed, this response only reached significance when compared with the control group in the 10 µg vaccine dose experiment (Fig. 7A, B).

We next sought to determine the contribution of CD4+ and CD8+T cells to the observed responses induced by the vaccine. To do this, we magnetically depleted either CD4+ or CD8+T cells from cell suspensions prepared from the spleens of mice vaccinated twice with 10 or 20 µg ß-S_13_EM-SARS-CoV-2 (Fig. 7E, F)(Supplementary Fig. 2B, C) and measured their IFN-γ responses to ß-S_13_EM-SARS-CoV-2 using ELISpot. While depletion of CD8 + T cells had no detectable effect on the size of the IFN-γ response to VLPs for either dose, CD4+ depletion reduced the frequency of spots to background levels in both cases (Fig. 7E, F)(Supplementary Fig. 2B, C, D), indicating that CD4+T cells were major contributors to the response elicited by VLP vaccination.

While the assay above clearly established that CD4+T cell responses were elicited by our vaccine, we wished to use more sensitive methods to determine whether any CD8+T cell responses were generated. To do this, we used an MHC-I tetramer containing the SARS-CoV-2 S protein peptide VNFNFNGL (S_539-546_)^46–49^ to stain splenocytes of mice vaccinated with two doses of 20 µg ß-S_13_EM-SARS-CoV-2 VLP/Addavax vaccine. We detected a small, but consistent response in these mice (Fig. 7G; Supplementary Fig. 2E; Supplementary Fig. 3 Lymphocyte gating strategy).

In a subsequent experiment, we enriched CD8+T cells from the spleens of vaccinated mice and performed ELISpot, restimulating with the SARS-CoV-2 peptide, or an unrelated peptide (SIINFEKL). Again, we detected a clear CD8 + T cell response in the VLP-vaccinated group (Fig. 7H). In summary, these experiments show that our ß-S_13_EM-SARS-CoV-2 VLP vaccine elicits a substantial T cell response predominantly driven by CD4+T cells, with some CD8+T cells also being generated.

### ß-S_13_EM-SARS-CoV-2/Addavax VLPs are protective against homologous challenge

**Fig. 8:**
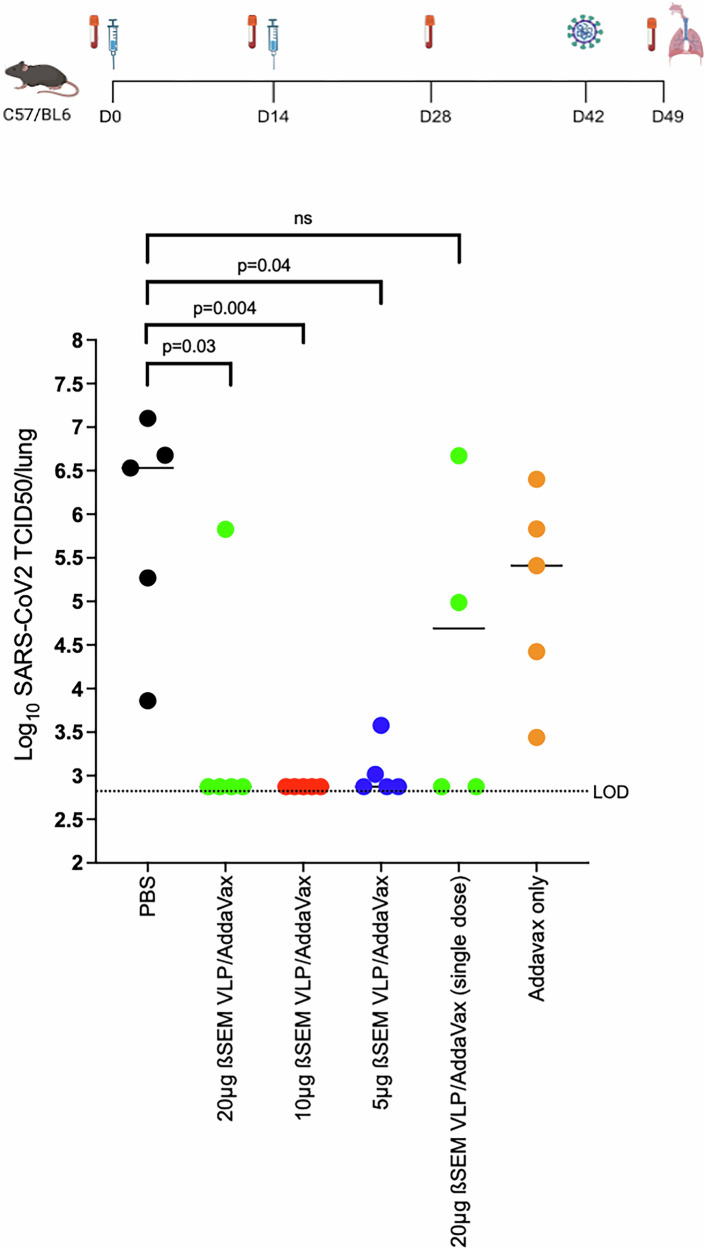
Determination of protection in a mouse model after immunisation with ß-SEM-SARS-CoV-2 VLP/Addavax. Titres of virus in lungs of mice (five per group) vaccinated subcutaneously with two doses 14 days apart with PBS, 20, 10 or 5 μg of ß-SEM-SARS-CoV-2 VLP/Addavax. Mice were aerosol challenged with ß-SARS-CoV-2 virus 28 days after the second immunisation. Age and sex matched PBS injected control C57/BL6 mice (*n* = 5) were also challenged. Three days after challenge, mice were euthanized and the titre of infectious virus (TCID_50_/ml) in lungs of individual mice were determined. Challenge experiments were performed twice and titrations repeated in triplicate. Means for each group are depicted. Data were analysed using one-way ANOVA and Tukey’s Multiple Comparisons test. LOD Limit of detection (Immunisation schedule figure created with BioRender.com).

Having shown that our ß-S_13_EM-SARS-CoV-2/Addavax vaccine produces strong humoral and T cell responses we sought to determine the protective efficacy of the vaccine against infection of lungs in a mouse SARS-CoV-2 challenge model using Beta-SARS-CoV-2 virus (Fig. 8). Mice were vaccinated twice, 2 weeks apart, with 5, 10 or 20 µg of ß-S_13_EM-SARS-CoV-2 VLPs mixed with Addavax or with a single dose of 20 µg of ß-S_13_EM-SARS-CoV-2 VLPs with Addavax and bled at days 0, 14 and 28. On day 42, mice were challenged with ß-SARS-CoV-2 variant virus. Complete protection was observed in mice immunised with 5 or 10 µg of ß-S_13_EM-SARS-CoV-2/Addavax vaccines (Fig. 8). Four of the five mice immunised with 20 µg ß-S_13_EM-SARS-CoV-2/Addavax had no detectable virus in their lungs (Fig. 8). Interestingly, only two mice immunised with a single dose of 20 µg ß-S_13_EM-SARS-CoV-2/Addavax vaccine were protected against challenge, with no detectable virus in their lungs (Fig. 8). This group of mice similarly demonstrated a more rapid decline in anti-RBD antibody responses (Fig. 5B).

### In vivo antibody responses to ß-S_13_EM-SARS-CoV-2/MF59 vaccine

**Fig. 9:**
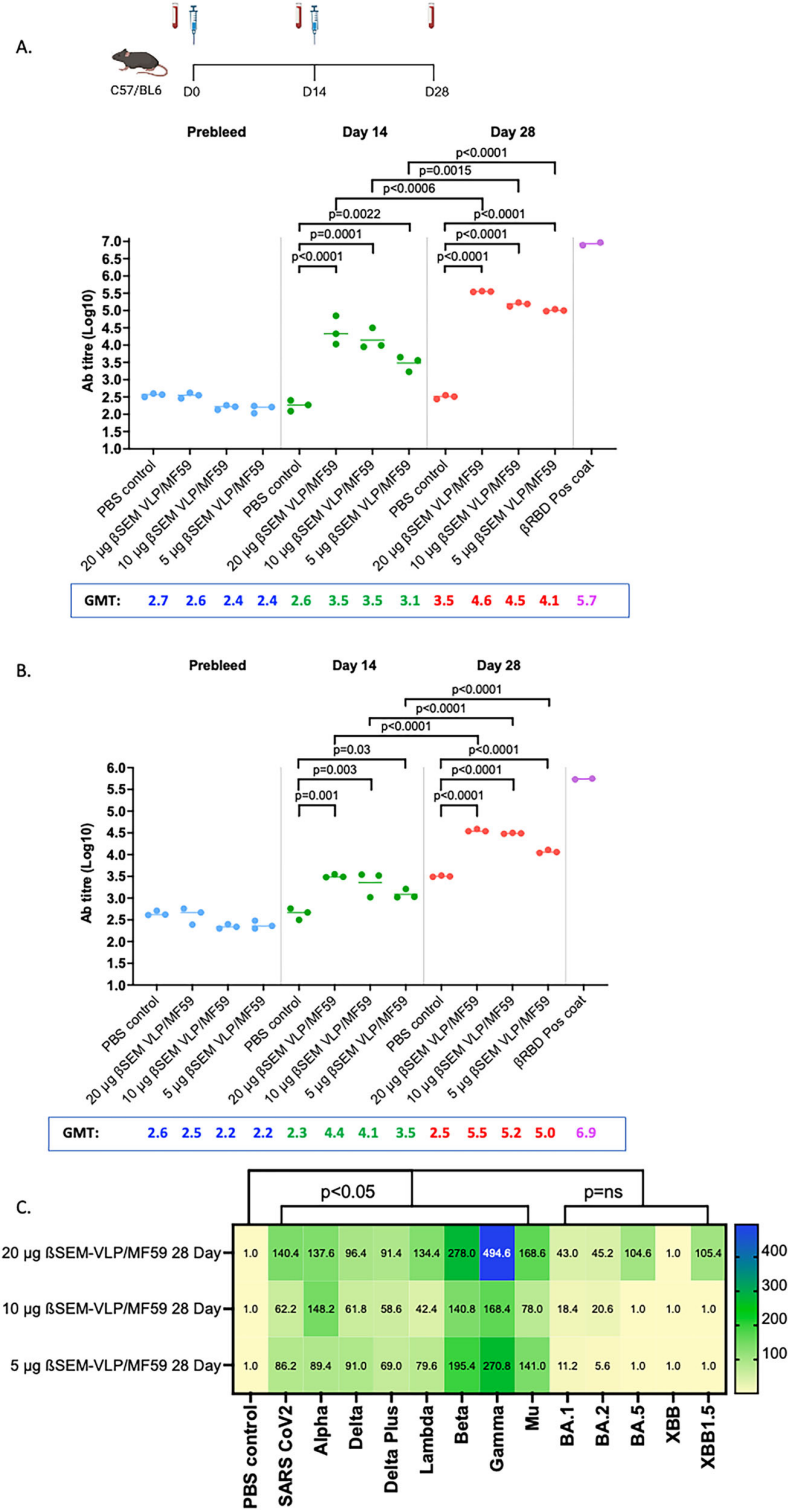
Antibody responses to ß-S_13_EM-SARS-CoV-2 VLP/MF59 vaccine. **A** Mice were immunised with 2 doses of 5, 10 or 20 µg of vaccine with or without MF59 2 weeks apart, bled at days 0, 14 and 28. Anti-ß-S_13_EM-SARS-CoV-2 VLP antibody responses were assessed using ELISA. Immunisations were performed twice, with 5 mice in each vaccine group. Sera for each group at respective time points were pooled and tested in triplicate. ELISAs were performed twice. Data from groups of mice were compared using one-way ANOVA and Tukey’s Multiple Comparisons test. Bar indicates mean value; each dot represents one mouse. Geometric mean titre (GMT) values are displayed for each group at day 0, 14 and 28. **B** Mice were immunised with 2 doses of 5, 10 or 20 µg of vaccine with MF59 2 weeks apart, bled at days 0, 14 and 28. Anti-ß-RBD antibody responses were assessed using ELISA, as before. Immunisations were performed twice with five mice in each vaccine group. Sera for each group at respective time points were pooled and tested in triplicate. ELISAs were performed twice. Data from groups of mice were compared using one-way ANOVA and Tukey’s Multiple Comparisons test. Geometric mean titre (GMT) values are displayed for each group at day 0,14 and 28. **C** Heat map of sNAb responses against SARS-CoV-2 variants. Samples from day 28 were tested in a multiplex sVNT inhibition assay for their ability to inhibit the binding to ACE2 to SARS-CoV-2 Alpha, Beta, Gamma, Delta, Delta+, Lambda, Mu, Omicron BA.1, BA.2, BA.5, XBB.1 and XBB.1.5. Multiplex assays were performed in duplicate. The mean titres of the half maximal inhibitory dilution (ID_50_) are shown for each sample and the range of titres shown in the legend bar. (Immunisation schedule figure created with BioRender.com).

We next sought to determine the antibody responses to ß-S_13_EM-SARS-CoV-2 formulated with MF59, an adjuvant approved for human use and that has been included in commercial influenza vaccines. All mice developed antibody responses as early as 14 days. Responses to ß-RBD were stronger than to ß-S_13_EM-SARS-CoV-2 VLPs. By Day 28 mice in all vaccine groups developed strong antibody responses to both ß-RBD and ß-VLPs (Fig. 9A, B; Table 2). Even mice immunised with 5 µg of ß-S_13_EM-SARS-CoV-2 VLPs developed antibody responses at day 28 that were almost as strong as with the 20 µg dose. Anti-ß-RBD geometric mean titres were highest in mice immunised with 20 µg of VLP vaccine (5.55; 95%CI, 5.25, 5.75)(Table 2).

**Table 2:** Geometric mean titers of anti-RBD antibodies in mice immunised with 5, 10 or 20 µg of ß-S_13_EM-SARS-CoV-2/MF59

	PBS	βSEM VLP 20 µg/MF59	βSEM VLP 10 µg/MF59	βSEM VLP 5 µg/MF59
Geometric mean	2.500	5.550	5.180	5.007
Geometric SD factor	1.023	1.002	1.011	1.006
Lower 95% CI of geo. mean	2.365	5.525	5.043	4.931
Upper 95% CI of geo. mean	2.642	5.575	5.320	5.083

The breadth of sNAb responses was again assessed using a multiplex RBD-ACE2 binding inhibition assay (Fig. 9C)^45^. Neutralising activity (ID_50_) against Alpha, Delta, Lambda, Beta, Gamma and Mu was observed in all groups of mice. The greatest breadth and highest sNAb GMT were observed in mice immunised with 20 µg ß-S_13_EM-SARS-CoV-2 VLPs with MF59 (Fig. 9C). Once again, the activity against Omicron BA.1, BA.2, BA.5, XBB and XBB.1.5 was negligible in all groups although at the highest dose of 20 µg ß-S_13_EM-SARS-CoV-2 VLPs/MF59, the vaccine produced sNAb against Omicron BA.5 and XBB.1.5 (Fig. 9C).

### ß-S_13_EM-SARS-CoV-2/MF59 vaccine is broadly protective against variants

**Fig. 10:**
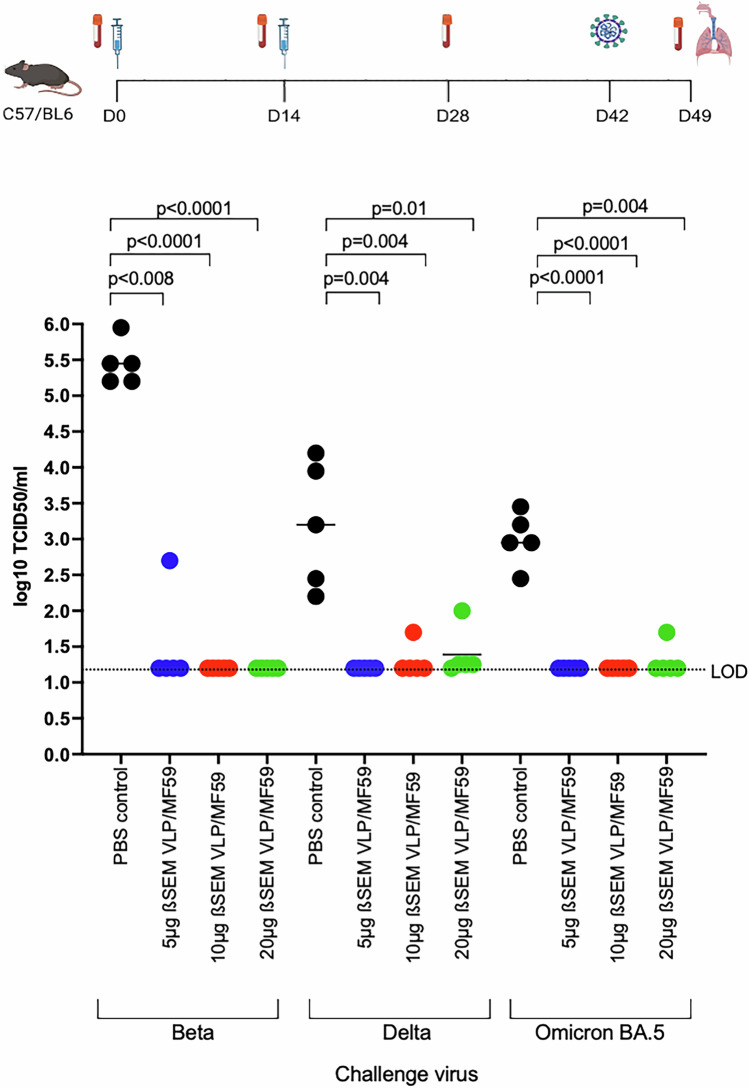
Determination of protection in a mouse model after immunisation with ß-SEM-SARS-CoV-2 VLP/MF59. Titres of virus in lungs of mice (five per group) vaccinated subcutaneously with two doses, 14 days apart, of 5, 10 or 20 μg of ß-SEM-SARS-CoV-2 VLP/MF59. To ensure mice could be infected with Omicron BA.5 and to maintain comparability across variant groups, mice were transduced intranasally with AAV-hACE2. Mice were challenged by intranasal inoculation with Beta-, Delta- or Omicron BA.5-SARS-CoV-2 viruses 28 days after the second immunisation. Age and sex-matched PBS vaccinated control C57/BL6 mice (*n* = 5) were also challenged. Three days after challenge, mice were euthanised and the titre of infectious virus (TCID_50_) in lungs of individual mice was determined in triplicate. Immunisation and challenge studies were performed twice. Means for each group are depicted. Data were analysed using one-way ANOVA and Tukey’s Multiple Comparisons test. LOD limit of detection. (Immunisation schedule figure created with BioRender.com).

We next sought to determine the protective efficacy of the ß-S_13_EM-SARS-CoV-2/MF59 vaccine against lung infection using Beta-, Delta- and Omicron BA.5 SARS-CoV-2 viruses. Omicron variants are known to replicate less efficiently in mice^50^. To overcome this, we used AAV-hACE2 to transduce mice with hACE2 rather than using K18-hACE2 transgenic mice to avoid ectopic expression of hACE2 in cells that do not normally express this transgene, specifically avoiding infection of the brain and to use a transduction model to more closely recapitulate infection in humans^51–54^. Mice were vaccinated twice, 2 weeks apart, with 5, 10 or 20 µg of ß-S_13_EM-SARS-CoV-2 VLPs formulated with MF59. On day 42, mice were challenged with the Beta, Delta- and Omicron BA.5 SARS-CoV-2 variants. All mice in each vaccine group, except for one mouse in the 5 µg group, were completely protected against lung infection with Beta-SARS-CoV-2 (Fig. 10). The one outlier in the 5 µg vaccine group was partially protected, with a lung viral titre that was 3 log_10_ lower than PBS control mice (Fig. 10). Mice in all vaccine groups were also completely protected against lung infection with both Delta- and Omicron BA.5 SARS-CoV-2 variants (Fig. 10). Interestingly, protection against Omicron BA.5 was maintained in all vaccine groups despite the absence of high titre anti-RBD NAb against ACE2 in the 5 and 10 µg dose groups (Fig. 9C). Analysis of viral titres in nasal turbinates showed robust replication of Beta-SARS-CoV-2 but not of Delta- and Omicron BA.5 SARS-CoV-2 variants in PBS control mice. Consequently, it was only possible to determine vaccine-induced protection in nasal turbinates against Beta-SARS-CoV-2. Vaccination produced up to a 2-log reduction in viral titres in nasal turbinates, with mice vaccinated with 5 or 10 µg of ß-S_13_EM-SARS-CoV-2 VLPs showing significantly lower titres than PBS control mice (Supplementary Fig. 4).

## Discussion

In this study, we have shown that our ß-SARS-CoV-2 VLP vaccine produces broad protective immune responses against SARS-CoV-2 variants, including Omicron BA.5. Interestingly, the breadth of protection achieved was not reliant on the vaccine only producing high titre NAb responses directed at blocking the RBD-ACE2 interaction. Both sVNT and multiplex RBD-ACE2 binding inhibition assays showed that the vaccine produces low-titre sNAb that target the RBD. In contrast, the microneutralisation assay did not show sNAb. However, we have previously shown that an ancestral-SARS-CoV-2 VLP vaccine adjuvanted with Addavax produced antibody responses that completely neutralised viral entry into organoid cultures based on air–liquid-interface (ALI)-differentiated human nasal epithelium (HNE)^39^ which may provide a more authentic model for both human SARS-CoV-2 infection and neutralisation^55^. The vaccine formulated with Addavax or MF59 produced strong total anti-RBD and anti-S antibody responses that may have also produced protection by binding neutralising and non-neutralising targets outside of RBD. However, the nature of these antibody responses and how they contribute to protection against SARS-CoV-2 variants warrants further investigation.

Several studies have shown that antibodies targeting the spike N-terminal domain (NTD), the S2 fusion loop^36,56–59^ and the stem helix of S2^60,61^ are able to neutralise SARS-CoV-2 and probably play an important role in protection. Our ß-SARS-CoV-2 VLPs are richly decorated in prefusion stabilised spikes that can deliver these protective targets in high density and may help to explain the protection observed against Omicron BA.5 in the absence of RBD-ACE2 NAb. Also, protection against SARS-CoV-2 infection can result from antibodies with Fc-effector functions like antibody-dependent cellular cytotoxicity (ADCC) and antibody-dependent cellular phagocytosis (ADCP) ^62^. It is possible that our VLP-based vaccine produces similar responses, thereby affording an additional mechanism of protection against infection. These potential modes of protection that our ß-SARS-CoV-2 VLP vaccine might produce warrant further investigation.

The ß-SARS-CoV-2 vaccine adjuvanted with MF59 produced complete protection against infection in lungs by Beta, Delta and Omicron BA.5 viruses. Although we were not able to show the same level of protection in nasal turbinates, the vaccine administered by the intramuscular route did produce significant reductions in ß-SARS-CoV-2 viral titres. Whilst this vaccine looks promising by producing broad protective responses, we are investigating the extent of cross-reactive antibody responses of the ß-SARS-CoV-2 VLP vaccine against a wide range of variants and whether Omicron BA.5, JN.1 and KP.3 SARS-CoV-2 VLP vaccines provide broader protection against current variants.

Existing COVID-19 vaccines have relied mostly on the delivery of the spike (S) protein or gene to produce S-specific humoral immune responses, including RBD NAb responses. Whilst these vaccines have been effective in producing targeted antibodies that neutralise virus, it has become evident that they are less able to neutralise and prevent infection with emerging SARS-CoV-2 variants^8,9,63–65^. For variants like Omicron BQ.1.1 and XBB.1, even three doses of either BNT162b2 or mRNA-1273 vaccines in the presence or absence of past Omicron BA.2 infection were up to 60-fold less effective at neutralising these variants^8^. In contrast, bivalent mRNA vaccines containing ancestral and Omicron BA.5 S genes have provided broader NAb responses against Omicron variants regardless of previous infection history^9^. Boosting individuals who had previously received 3 doses of BNT162b2 with a dose of bivalent BA.5 vaccine produced a 5- to 8-fold rise in geometric mean neutralising titre for variants BA.4/5-, BF.7-, BA.4.6-, BA.2.75.2- and BQ.1.1, compared to a 4^th^ dose of monovalent BNT162b2 vaccine. However, for XBB.1 this was only a modest 2- to 3-fold rise^9^. These reports highlighted the importance of developing more effective vaccines to keep abreast of emerging SARS-CoV2 variants, like Omicron EG.5, 2.86, JN.1, KP.3 and NB.1.8.1, that have enhanced transmissibility and ability to escape vaccine-induced immunity. To advance vaccines, new approaches may need to encompass technologies that incorporate additional viral structural proteins and not spike alone to deliver broader protective immune responses that are not mostly reliant on producing anti-RBD NAb responses. Our SARS-CoV-2 VLP vaccine contains S, M and E proteins, with E correctly assembled as a dimer^41^. With the adaptability of our platform in being able to readily alter the S protein presented, SARS-CoV-2 VLPs provide an alternative vaccine platform that can offer protection against emerging variants.

Several studies have highlighted the importance of the role of viral M, nucleoprotein (N) and E proteins in producing protective and memory CD4+ and CD8+T cell responses to coronaviruses^25–27,29,66^. In addition, persistent SARS-CoV-2 specific T cell responses are preserved against emerging variants and appear to be an central component to providing protection against severe disease^28,67,68^. The inclusion of these proteins in a vaccine will be an important requirement for the development of a more broadly effective vaccine against emerging SARS-CoV-2 variants that will not only prevent infection, but that may also prevent the development of severe disease^69^.

Our vaccine produced both CD4+ and CD8 + T cell responses, although the former made up the predominant T cell response. This is nonetheless an important finding as CD4+ and CD8 + T cell responses play a critical role in providing protection against SARS-CoV-2^25,26,29,70^. However, the protective role of both M and E specific T cell responses produced by our vaccine requires further investigation. In addition, COVID vaccines are also able to produce CD4+ and CD8 + T cell responses that retain activity against a broad range of variants spanning Alpha through to Omicron that may provide protection against reinfection^28^. The ability of our vaccine to produce similar T cell responses is important for the continued development of a VLP-based vaccine as SARS-CoV-2 variants continue to emerge globally.

The rapid waning of antibody response encountered with current mRNA vaccines and natural infection is problematic, as it has necessitated frequent vaccine boosting. The loss of antibody over time may be a reflection of the inability of these vaccines to produce long-lived plasma cells (LLPC), despite stimulating the production of memory B cells, germinal centre responses and bone marrow plasma cells but not with phenotypic markers consistent with LLPC^71–75^. An important component of driving a durable antibody response and the generation of LLPC following vaccination relates to the ability of a vaccine to deliver multivalency in antigen presentation thereby ensuring efficient cross-linking of B cell receptors (BCR) that is essential for the development of memory B cell responses that lead to the formation of LLPC^76–78^. This is also important for the recruitment of T cell help for the generation of memory B cell responses^79^. Particulate antigens, like VLPs, are well suited to producing potent B cell responses and have significant advantages over soluble antigens of low valency or mRNA vaccines, which may not be able to simulate the multivalency of particulate protein-based vaccine antigens that present efficiently to B cells or antigen-presenting cells^80,81^. It will be important to show whether the ß-SARS-CoV-2 VLP vaccine is able to produce LLPC that may correlate with durable antibody responses.

The electron microscopy and immunogold analyses of our ß-SARS-CoV-2 VLPs show that the particles contain a large number of surface spikes. These have been stabilised into a prefusion conformation and so should be able to present protective epitopes efficiently. It is interesting to note that studies that have explored the molecular architecture of SARS-CoV-2 viruses have shown that on average an intact virion contains 25 to 50 prefusion spikes that are randomly distributed across the surface of the virus^82^. It is possible that the high density of prefusion spikes on the ß-SARS-CoV-2 VLPs might result in a more efficient cross-linking of BCRs, thereby producing stronger and longer lasting antibody responses. This may explain why our SARS-CoV-2 VLP vaccines are able to produce strong and durable antibody responses in mice^24^. We have also previously shown that a quadrivalent VLP vaccine against hepatitis C similarly produces durable antibody responses^83^. Whether the ß-SARS-CoV-2 VLPs achieve this interaction and potentially drive the development of LLPC remains an important question that needs to be further investigated. If SARS-CoV-2 VLPs produce LLPC, this may offer an effective solution to the short-lived antibody responses observed with mRNA vaccines.

We administered our vaccine by intramuscular injection and although vaccination was protective against lung infection, it only produced modest reductions in viral titres in nasal turbinates. Several recent studies have shown that the intranasal administration of RBD, RBD dimer and adenoviral vectored spike vaccines produces strong mucosal immune responses and reductions in viral titers in nasal turbinates that are comparable to the effects in lungs^84–86^. As a next step, it will also be important to study intranasal administration of our ß-SARS-CoV-2 VLP vaccine to determine its ability to produce stronger protective mucosal immune responses.

We have developed an effective ß-SARS-CoV-2 VLP vaccine that produces strong antibody and T cell responses and that is broadly protective against SARS-CoV-2 Beta, Delta and Omicron BA.5 variants. Having also developed methodologies for producing and purifying our SARS-CoV-2 VLP vaccine to large scale will be of significant interest to industry and offers a viable alternative to mRNA vaccines that warrants further investigation in clinical trials in humans.

## Methods

### Production and purification of ß-SEM-SARS-CoV-2 VLPs

A synthetic ß-SEM-SARS-CoV-2 gene construct of 4.8 kb (NCBI Reference Sequence: NC_045512.2) that encodes a polyprotein of the structural proteins ß-Spike (3.831 kb), Envelope (225 bp) and Membrane (680 bp) (GeneArt, Thermofisher Scientific, USA) was synthesised with the endoplasmic reticulum (ER) signal peptidase/signal peptide peptidase (SP/SPP) sequence between the S, E and M gene. The construct supplied was resuspended in sterile MilliQ water to a concentration of 0.25 µg/µL. The method for producing the recombinant adenoviruses expressing SARS-CoV-2 S, E and M genes has been described previously^24,39^. This recombinant adenovirus was designated rAd-ß-SEM-SARS-CoV-2.

In a second approach, the VLPs were produced using two recombinant adenoviruses. The first contained the ß-S gene, amplified from the full-length ß-SEM construct and subcloned into the AdEasy adenoviral expression system as described previously^24,39^. This recombinant adenovirus was designated rAd-ß-S-SARS-CoV-2. The second contained the Wuh-EM genes that were amplified from a synthetic Wuh-SEM-SARS-CoV-2 gene construct that has an SP/SPP sequence separating the E and M genes and that we have reported previously^24^. This recombinant adenovirus was designated rAd-E_SPP_M-SARS-CoV-2. The primer sequences for the amplifications are shown in Supplementary Table 1.

### Infection of Vero cells and determination of recombinant adenoviral titres

Adapted Vero cells (2% foetal bovine serum, FBS) were infected with (1) rAd-ß-SEM-SARS-CoV-2 or (2) rAd-ß-S-SARS-CoV-2 together with rAd-E_SPP_M-SARS-CoV-2 viruses as described previously^24,39^. Virus titre for each sample determined as 50% tissue culture infectious dose (TCID_50_/mL) by limited dilution using the Kärber method, as described previously^39^. The production of ß-SEM-SARS-CoV-2 VLPs has been described previously^24,39^. In brief, VLPs were produced using 5 stack cell factories (Corning Life Sciences, USA). Each layer of the cell factory has a growth surface area of 175 cm^2^, thereby providing a total of 875 cm^2^ per cell factory. Vero cells (4.9 × 10^6^) were seeded into each 175 cm^2^ layer (2.45 × 10^7^ cells per 5-stack cell factory) in 20 ml per layer of OPTIPRO SFM (Gibco, Thermofisher Scientific, USA) supplemented with 2% Glutamax (Gibco, Thermofisher Scientific, USA), 1% penicillin and streptomycin and incubated overnight at 37 °C in 4% CO_2_. Once the Vero cells had grown to 80% confluency (18.6 × 10^6^ cells), the monolayers were infected with respective recombinant adenoviruses each at an MOI of 1.0. For production of one batch of ß-SEM-SARS-CoV-2 VLPs, five cell factories were prepared and VLPs purified from a total cell culture supernatant volume of 500 ml.

### Purification of VLPs from Vero cell factories

Supernatant was collected from each multilayer cell factory and clarified at 3750 *g* for 10 min at 4 °C. From this point, all procedures were performed at 4 °C or on ice. Supernatant was collected after clarification and centrifuged at 3750 *g* for 30 min at 4 °C. The supernatant, containing VLPs, was then collected and purified by a sequential process on tangential flow filtration (TFF), diafiltration followed by anion exchange chromatography. ß-SARS-CoV-2 VLPs were then concentrated Amicon ultrafiltration and finally sterilised through a 0.45 µm filter. The VLP samples were quantified using the Bradford assay (Thermofisher # 23200) on a Nanodrop 2000 and stored at −80 °C in 60 µg aliquots.

ß-SARS-CoV-2 VLPs harvested from culture supernatants were analysed by Western blot. Following separation by SDS-PAGE and transfer to polyvinylidene fluoride (PVDF) membrane, blots were probed with anti-S (40591-T62, Sino Biologicals), anti-M (MBS434281, MyBioSource) and anti-E (MBS8309656, MyBioSource) antibodies for SARS-CoV-2 VLPs as described previously^24,39^.

### Fluorescence microscopy

Vero cells were maintained in DMEM (Thermo Fisher) supplemented with 2% FBS (GIBCO), 1×GlutaMAX, (Thermo Fisher), 60 µg/mL Penicillin (BenPen^TM^, CSL/Seqirus) and 100 µg/mL Streptomycin (Sigma). Cells were seeded into 4 × 24 well plates at a density of 5 × 10^4^ cells per well in duplicate for infection the following day. Virus dilutions of the adenovirus SARS-CoV-2 construct were added to the wells. Virus was first diluted in 600 µL of media, added to the wells at 1 in 8, serially diluting two-fold and incubated for 4 h before adding 1.2 mL media. Green fluorescence protein (GFP) was observed over 6 days under a fluorescence microscope. The dilution associated with a near 100% infection rate by GFP expression and the highest cell viability was used for further experiments.

### Electron microscopy

Prior to performing immuno-gold electron microscopy (IEM), the quality of the samples was verified using a negative staining procedure, with subsequent transmission electron microscopy (TEM) examination. IEM was performed on copper 400-mesh glow-discharged formvar-carbon coated grids with ß-S_13_EM-SARS-CoV-2 VLPs and staining with polyclonal rabbit anti-SARS-CoV-2 RBD antibody (MBS2563840, My BioSource).

Negative stains were prepared by directly applying 6 µL of ß-SARS-CoV-2 VLP containing suspension or SARS-CoV-2 AUS/VIC01/2020 control material to a glow-discharged 400-mesh copper formvar-carbon coated grid and allowed to adsorb for 20 s. The suspension was removed by blotting and then negatively stained using 3% phosphotungstic acid (pH 7.0). After negative staining, the grids were blotted again to remove excess stain and air-dried at room temperature.

For IEM, copper 400-mesh glow-discharged formvar-carbon coated grids were placed onto a 10 µL drop of the ß-S_13_EM-SARS-CoV-2 VLP suspension or SARS-CoV-2 control and allowed to adsorb for 5 min before being washed three times in 10 mM HEPES/saline buffer (HN buffer). Washed grids were transferred to a 25 µL drop of HN buffer containing 1% bovine serum albumin (BSA) and incubated in a room temperature humidified chamber for 20 min. The grids were then blotted dry and immediately transferred to a 25 µL drop of HN buffer containing 0.2% BSA and polyclonal rabbit anti-SARS-CoV-2 RBD antibody (MBS2563840, My BioSource) diluted 1:500, then incubated for 1 h in a room temperature humidity chamber. After incubation, the grids were washed three times in HN buffer containing 0.2% BSA and transferred to a 25 µL drop of HN buffer containing 0.2% BSA and monoclonal anti-rabbit antibody conjugated with 10 nm gold nanoparticles at a dilution of 1:20, then incubated for 1 h in a room temperature humidified chamber. Grids were washed five times in HN buffer containing 0.2% BSA followed by three rinses in 0.22 µm syringe-filtered MilliQ H_2_O. The grids were then negative-stained using 3% phosphotungstic acid (pH 7.0). The negative-stained grids were blotted to remove excess stain and air-dried at room temperature. Negative stained grids were examined using an FEI Tecnai T12 Spirit electron microscope operating at an acceleration voltage of 80 kV. Electron micrographs were collected using an FEI Eagle 4k CCD camera. File type conversion and morphometry were performed using the FEI TIA software package.

### ELISA assay for determination of antibody reactivity of ß-S_13_EM-SARS-CoV-2 VLPs

Purified ß-S_13_EM-SARS-CoV-2 VLPs were tested by coating 96-well, flexible, flat-bottomed PVC microtiter plates (442404, Nunc, USA) with 50 µL of ß-S_13_EM-SARS-CoV-2 VLP and ELISAs performed as described previously^24,39^.

### Immunoprecipitation

VLPs were immunoprecipitated with a murine RBD-specific monoclonal antibody and detected with a rabbit anti-spike polyclonal antibody (40591-T62, Sino Biologicals, China) essentially as previously described^87^. Briefly, ß-S_13_EM-SARS-CoV-2 VLPs were incubated at 4 °C overnight with murine anti-RBD monoclonal antibody (clone 20-14-5) coupled to protein A sepharose, (or murine anti-SARS-CoV-2 nucleoprotein monoclonal antibody as negative control), centrifuged at 17,000 *g* for 5 min at 4 °C and the pellets washed twice (0.5 ml 0.05% Triton X-100, 10 mM Tris-HCl (pH 7.5), 2 mM EDTA in 150 mM NaCl which was subsequently decreased to 0.5 mM NaCl) and a final wash in 10 mM Tris-HCl, pH 7.5. The pellet was collected, resuspended in 20 µl Laemmli buffer, heated to 100 °C for 2 min, separated by 4–12% SDS-PAGE, transferred to PVDF and probed with rabbit anti-Spike polyclonal antibody.

### Mice

C57BL/6 mice were bred in-house at the Peter Doherty Institute for Infection and Immunity, Biological Research Facility, Melbourne, Australia. 6–10-week-old male mice were used for SARS-CoV-2 immunisation studies. All procedures were approved by the University of Melbourne Animal Ethics Committee (AEC 2023-20198-42712-15).

### In vivo experiments

Mice were immunised intramuscularly with two doses of 5, 10 or 20 µg of ß-S_13_EM-SARS-CoV-2 VLP, formulated with AddaVax (vac-adx-10, Invivogen, USA), MF59 (CSL Australia) or PBS alone. Mice were either (1) immunised on days 0 and 14, bled at days 0, 14 and euthanized on days 28 or 42 or (2) for T cell studies, on days 0 and 14, bled at days 0 and 14 and euthanized on day 21.

For the virus challenge in experiments using AddaVax as adjuvant, mice were vaccinated at days 0 and 14 with PBS (negative control), 5, 10 or 20 μg of ß-S_13_EM-SARS-CoV-2 VLP with AddaVax (1:1 v/v) in a total volume of 50 μL. Blood was collected at day 0, 14, 28 and 42. At day 45, mice were challenged with ß-SARS-CoV-2 virus and at day 35 euthanized and spleens collected.

ß-SARS-CoV-2 infection was performed using an inhalation exposure system (Glas-Col, LLC, Terre Haute, IN, USA) loaded with 1.5 × 10^7^ SARS-CoV-2 TCID_50_ as described previously^39^. Briefly, animals were placed in compartmented mesh baskets within the chamber of a Glas-Col Inhalation Exposure System and exposed to nebulised ß-SARS-CoV-2 virus for 30 min. All procedures involving animals and live SARS-CoV-2 were conducted in an OGTR-approved Physical Containment Level 3 (PC3) facility at the Walter and Eliza Hall Institute of Medical Research (Cert-3621) and were approved by the Walter and Eliza Hall Institute of Medical Research Animal Ethics Committee (2020.016).

For virus challenge experiments using MF59 as adjuvant, mice were vaccinated at days 0 and 14 with 5, 10 or 20 μg of ß-S_13_EM-SARS-CoV-2 VLP with MF59 or PBS (negative control), in a total volume of 50 μL. Bleeds were taken at day 0, 14 and 28. At day 30, vaccinated mice were transferred to the Physical Containment Level 3 (PC3) facility AgriBio, Centre for AgriBioscience, Bundoora, Australia for SARS-CoV-2 infections. Mice were lightly anaesthetised with isoflurane and transduced intranasally with AAV-hACE2 in a volume of 75 µl in order to broaden the ability to infect mice with Omicron variants. At 4-days post-transduction, mice were lightly anaesthetised with isoflurane and inoculated intranasally with 1 × 10^4^ TCID_50_ of either SARS-CoV-2/Australia/Vic/61994 (BA.5) (Omicron), SARS-CoV-2/Australia/QLD/1520/2020 (B.1.351) (Beta) or SARS-CoV-2/Australia/Vic/18440/2021 (B.1.617.2) (Delta) in a volume of 50 µl, as described previously and elsewhere^52,88^. Mice were euthanized at 3 days post-infection and organs were harvested for titration. Lungs and nasal turbinates were homogenised in 2 mL and 1 mL of PBS, respectively, and stored at −80 °C until titration.

Serum was used for the evaluation of antibodies by ELISA, ß-S_13_EM-SARS-CoV-2 VLP, or ß-RBD protein as coating antigen. Splenocytes were collected for the analysis of the T cell responses in mice immunised with VLPs by flow cytometry and ELISpot assay, respectively.

### Preparation of splenocytes

Spleens were placed into Petri dishes with 0.5% PBS/BSA and macerated with the plunger of a 5cc syringe. Homogenate was then transferred to a 70 µm strainer placed over a 50 ml tube. RF10 media (RPMI 1640 medium (61870127, Thermo Fisher Scientific), 10% foetal calf serum (10099141, qualified, Australia), 7.5 mM HEPES, 76 µM 2-mercaptoethanol (M6250, Sigma, USA), 150 U/mL penicillin, 150 mg/mL streptomycin (Sigma),150 µM non-essential amino acids (11140050, Thermofisher Scientific) was added slowly, up to 12 mL while using the plunger of syringe to squeeze/macerate the spleen through the strainer. The homogenate was centrifuged at 500 × *g* for 7 min at 4 °C and decanted. Four mL of Hybri-max Lysis Buffer (R7757, Sigma, USA) was added and incubated for 1 min at room temperature with gentle mixing. This was spun down again at 500 g for 7 min at 4°C and decanted. The cells were resuspended gently in cold PBS and centrifuged at 500 × *g* for 7 min and then resuspended in 15 ml RPMI and counted using the trypan blue method. For ELISpot assay (described below), cells were then transferred to 4 wells of a 12 well plate at 1 mL/well, 5 × 10^6^ cells/well and topped up with RF10 (supplemented with 10 ng/mL interleukin 2 (IL-2)). Twenty micrograms of ß-S_13_EM-SARS-CoV-2 VLP was then added to 2 wells for stimulation and the plate was incubated for 5 days in a 37 °C humidified incubator with 5% CO_2_.

### ELISA assay to determine anti-VLP- and RBD-specific antibody responses following immunisations with ß-S_13_EM-SARS-CoV-2 VLPs

ELISA plates were coated with 50 µL/well of ß-S_13_EM-SARS-CoV-2 VLP at 20 µg/ml or ß-RBD at 5 µg/mL in PBS and incubated at 4 °C overnight in a humidified chamber and tested against immune sera as described previously^39^. The coating antigens were then discarded, and wells blocked with 100 µL of 2% BSA/PBS for 1 h at 37 °C in a humidified chamber. Serum from immunised mice was initially diluted 1 in 10 in blocking buffer, then serially diluted. Fifty microlitres of each serum dilution was added to coated wells. Plates were incubated for 1 hr at room temperature and wells were then washed four times with PBS with 0.05%Tween20. Fifty microlitres of anti-mouse antibody conjugated to HRP (AB97046, Abcam, USA) diluted 1:10,000 was then added to wells and incubated at room temperature for 1 h. Plates were then washed four times with PBS with 0.05% Tween20 and developed by adding 50 µL of Tetramethylbenzidine (TMB) substrate (002023, Thermo Fisher Scientific) and stop solution as described above. Absorbance values were determined on a BMG Labtech Microplate Reader (BMG, Germany) at 450 nm.

### SARS-CoV-2 surrogate virus neutralization test (sVNT) assays

Assays were performed using an sVNT Kit (GenScript #L00847-A; wild type RBD-HRP) following manufacturer’s protocol. Percentage of inhibition was calculated according to the following formula:

$$\% {\rm{of\; inhibition}}=\,1-\frac{{\rm{OD\; value\; of\; sample}}}{{\rm{OD\; value\; of\; negative\; control}}}\times 100\, \%$$

### Multiplex sVNT assay

Multiplex sVNT against 13 different SARS-CoV-2 viruses, including Wuh-1, Alpha, Delta, Delta-Plus, Lambda, Gamma, Beta, Mu, Omicron BA.1, BA.2, BA.5, XBB.1 and XBB.1.5 was performed as previously described^45^. Briefly, Luminex avidin MagPlex microspheres coated with biotinylated RBD proteins were incubated with serial dilutions of sera from vaccinated mice for 60 min before the addition of R-Phycoerythrin conjugated human ACE2, followed by a further 60 min incubation. The binding or loss of binding of ACE2 was measured as MFI by the Luminex MagPix instrument. Results are expressed as neutralisation titre 50%.

### SARS-CoV-2 microneutralization

Vero/TMPRSS2 cells were seeded in 96-well plates at 10^4^ cells/well. The next day, serum samples were heat-inactivated at 56 °C for 30 min and then serially diluted (twofold, starting at 1:10) in DMEM (HyClone, 11965-084) plus 10% FBS in 96-well plates. Approximately 100 focus-forming units (FFU) of each SARS-CoV-2 strain was added to each well and the serum plus virus mixture was incubated for 1 h at 37 °C. Following co-incubation, the medium was removed from the cells and the serum sample plus virus mixture was added to the cells which were incubated 37 °C and 5% CO_2_ for 24 h. Cells were fixed with 10% neutral buffered formalin (Sigma, HT501128) for 1 h at 4°C and processed for immunofluorescence microscopy. Briefly, cells were permeabilized with 0.1% Triton X-100 (v/v) in 0.2% BSA/PBS (w/v) for 10 min, blocked in 0.2% BSA/PBS for 10 min and labelled with 1 μg/mL of rabbit anti-SARS-CoV nucleocapsid antibody (Sinobiological #40143-R004) for 1 h, all at room temperature. After three washes with 0.2% BSA/PBS, cells were incubated with goat anti-rabbit antibody conjugated to Alexa Fluor (AF) 488 (Invitrogen, A-11008) diluted at 1:2000 in 0.2% BSA/PBS for 45 min at room temperature. Samples were washed 3× and labelled with Hoechst 33342 (Invitrogen, H3570) for 10 min to visualise nuclei. Cells were imaged on a Nexelon Celigo high content imager and analysed for percentage infection based on AF488 positive cells divided by total number of nuclei. The neutralisation titres were calculated relative to a virus-only control (no serum) set at 100%, using GraphPad Prism 9.1.2 (La Jolla, CA) default nonlinear curve fit constrained between 0 and 100%.

### ELISpot and isolation of primary dendritic cells

The methods for IFN-γ ELISpot, CD4+ and CD8+T cell depletion and the isolation of Dendritic cells from the spleens of naïve mice, or mice treated with FMS-like tyrosine kinase 3 receptor ligand (Flt3-L)-producing cells have been described previously^24,39^.

### Measurement of viral loads via 50% tissue culture infectious dose (TCID_50_)

Titration was performed in flat-bottom 96-well plates (1.75 × 10^4^ Vero cells/well) as previously described^89^. Supernatant from homogenised lungs was added to wells and serially diluted. Cells were incubated for 4 days, until virus-induced cytopathic effect (CPE) was recorded. Titre (TCID_50_/mL) was calculated using the Spearman and Kärber algorithm.

### Institutional Review Board Statement

The study was conducted in accordance with the Medicine and Dentistry Human Ethics Sub-Committee, University of Melbourne (HREC/2057111). The animal study protocol was approved by The Walter and Eliza Hall Institute of Medical Research Animal Ethics Committee (2020.016) and the University of Melbourne Animal Ethics Committee (2020-20198).

## Supplementary information

Supplementary Information

## Data Availability

The data that support the findings of this study are not openly available due to reasons of commercial sensitivity but are available from the corresponding author upon request. Data are located in controlled-access LabArchives data storage at the University of Melbourne.
