# Supplementary material for: Preclinical development of a cross-protective β-SARS-CoV-2 virus-like particle vaccine adjuvanted with MF59
Source: NPJ Vaccines. 2026 Jan 17;11:34. doi: 10.1038/s41541-025-01355-y (PMC12858910; doi:10.1038/s41541-025-01355-y)
Supplement: Supplementary file 1 — Supplementary Information [file 41541_2025_1355_MOESM1_ESM.pdf]

Supplementary information

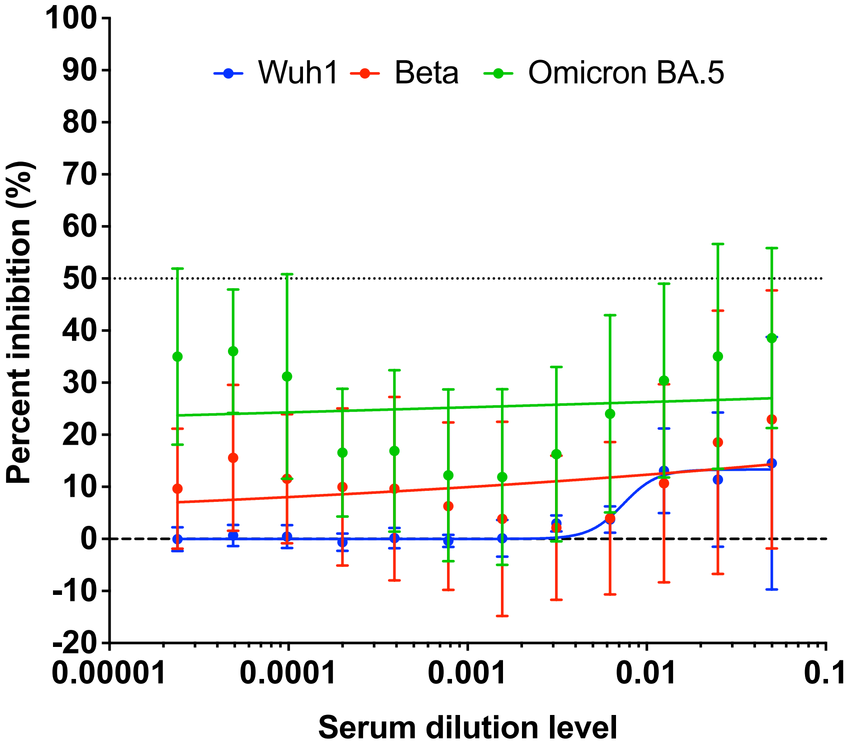

Suppl Figure 1. Microneutralisation assay. Two groups of 5 C57BL/6 mice were immunized with two doses of 10µg of ß-SARS-CoV-2 VLP vaccine adjuvanted with Addavax, 14 days apart. Mice were bled on days 0, 14 and euthanised on day 28. Sera from day 28 were tested by microneutralisation assay against Ancestral (Wuh1), Beta (B.1.351) and Omicron BA.5 SARS-CoV-2 viruses.

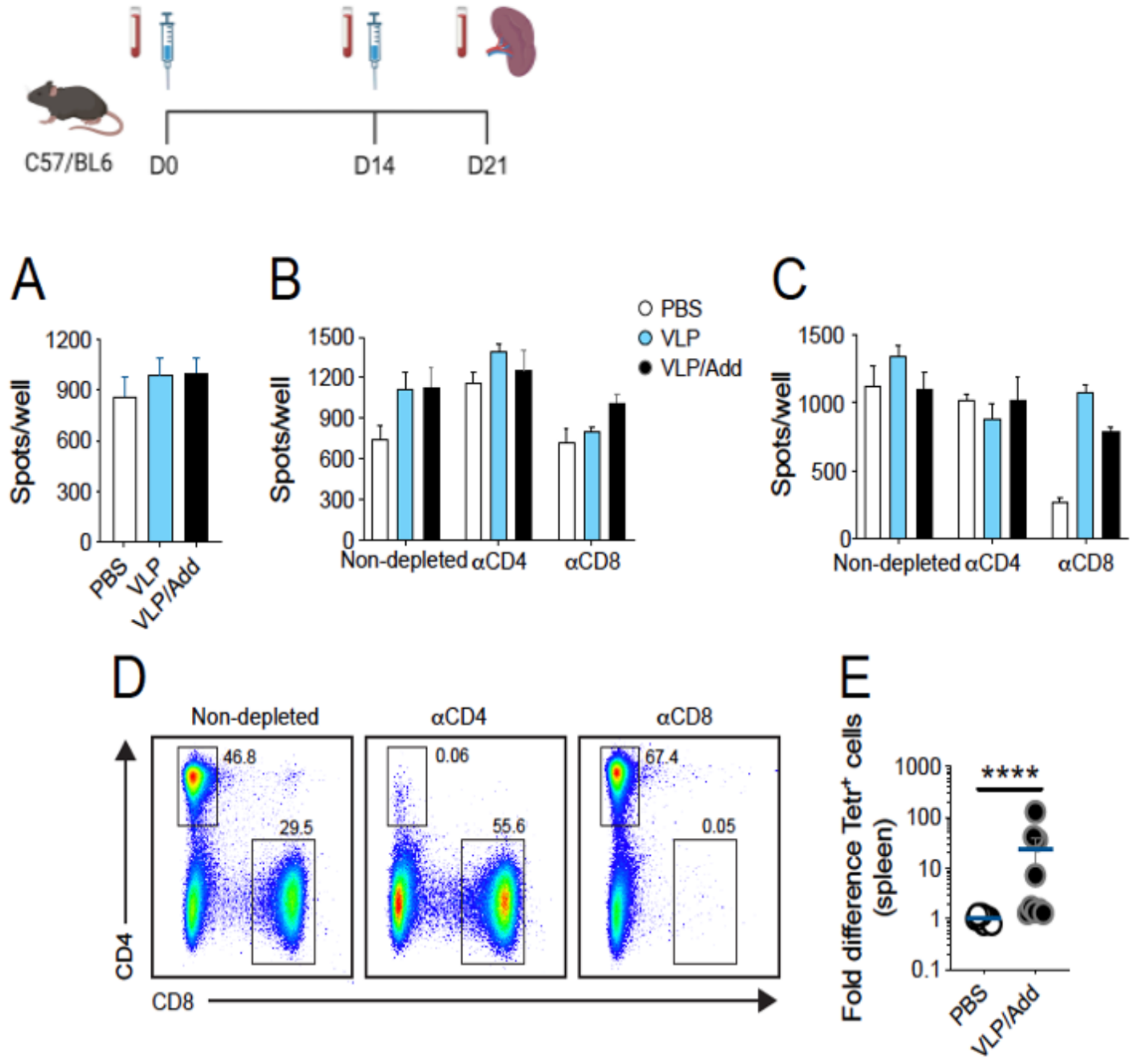

Suppl Figure 2. A. Responses to the positive control, concavalin A (5µg/mL), by splenocytes in 1A. **B.** Responses to the positive control, concavalin A (5µg/mL), by splenocytes in 1E. **C.** Responses to the positive control, concavalin A (5µg/mL), by splenocytes in 1F. **D.** Depletion efficiency in 1E, measured by FACS by gating on $CD19^-$ splenocytes and plotting $CD4^+$ vs $CD8^+$ cells. **E.** Pool of 3 experiments done as in 1G. Numbers of $S_{539\text{-}546}$ tetramer positive CD8 T cells are shown as a fold difference between ß-S_{13}EM-SARS-CoV-2 VLP/Addavax vaccinated mice vs PBS injected controls. Data were compared using a Mann-Whitney test.

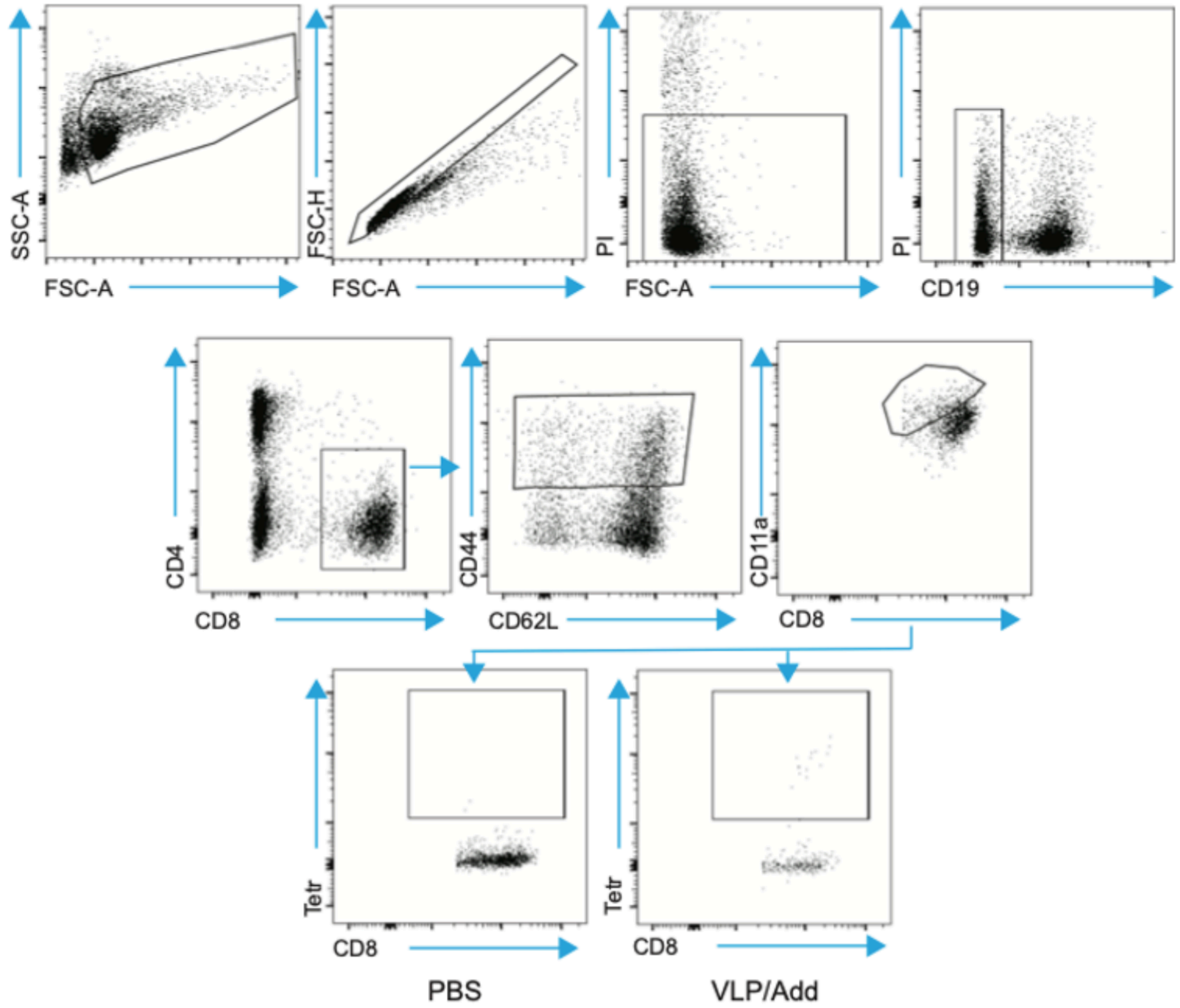

Suppl Figure 3. Lymphocytes were gated as single, live cells (propidium iodide[PI]-negative), non-B cells (CD19-negative), CD8-positive, activated T cells (CD44-positive, CD11a-high and CD8-low) and tetramer-positive.

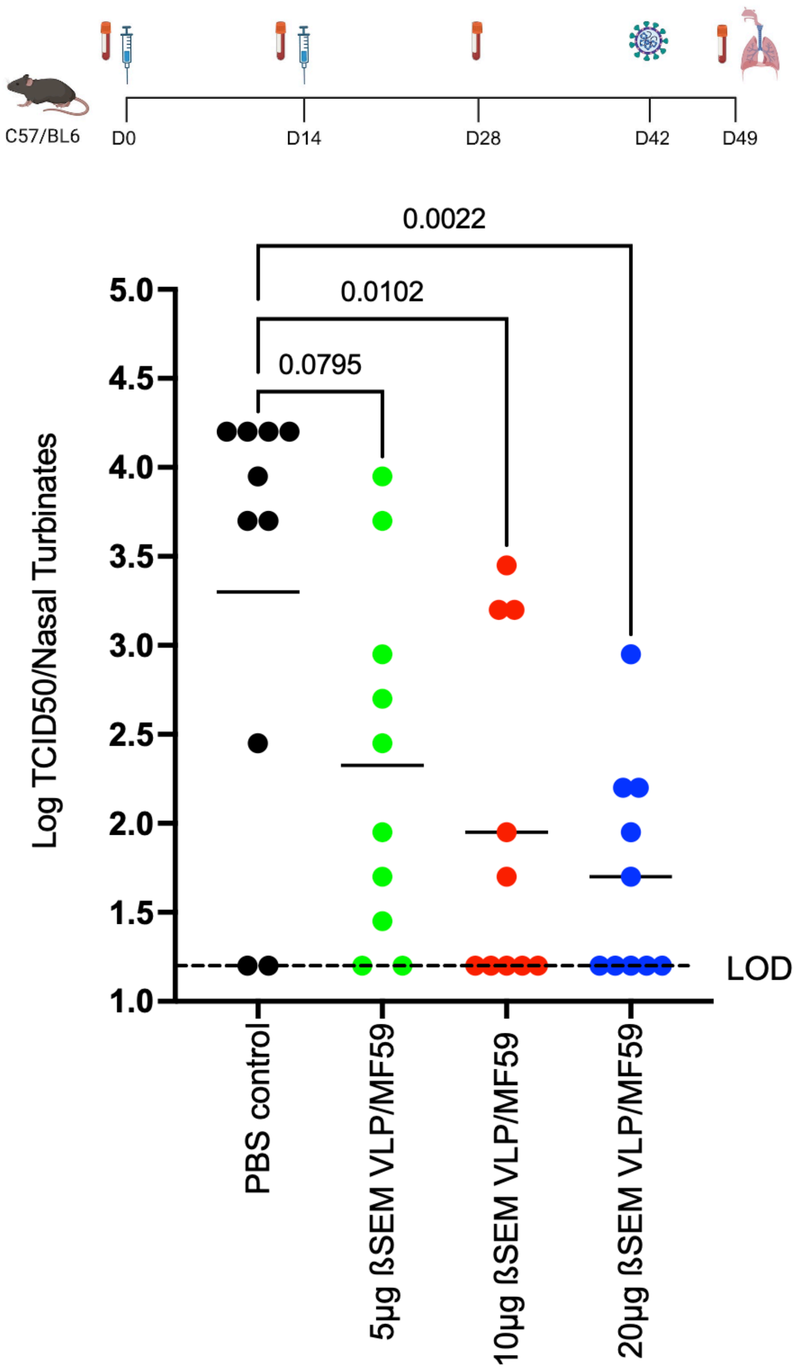

Suppl Figure 4. Determination of protection in a mouse model after immunization with ß-SEM-SARS-CoV-2 VLP/MF59. Titres of virus in nasal turbinates of mice (five per group from two separate infections) vaccinated subcutaneously with two doses, 14 days apart of 5, 10 or 20µg of ß-SEM-SARS-CoV-2 VLP/MF59. Mice were challenged by intranasal inoculation with Beta-SARS-CoV-2 viruses 28 days after the second immunisation. Age and sex matched PBS vaccinated control C57/BL6 mice (n = 5) were also challenged. Three days after challenge, mice were euthanised and the titre of infectious virus ($TCID_{50}$) in nasal turbinates of individual mice was determined in triplicate. Immunization and challenge studies were performed twice. Means for each group are depicted. LOD: Limit of detection.

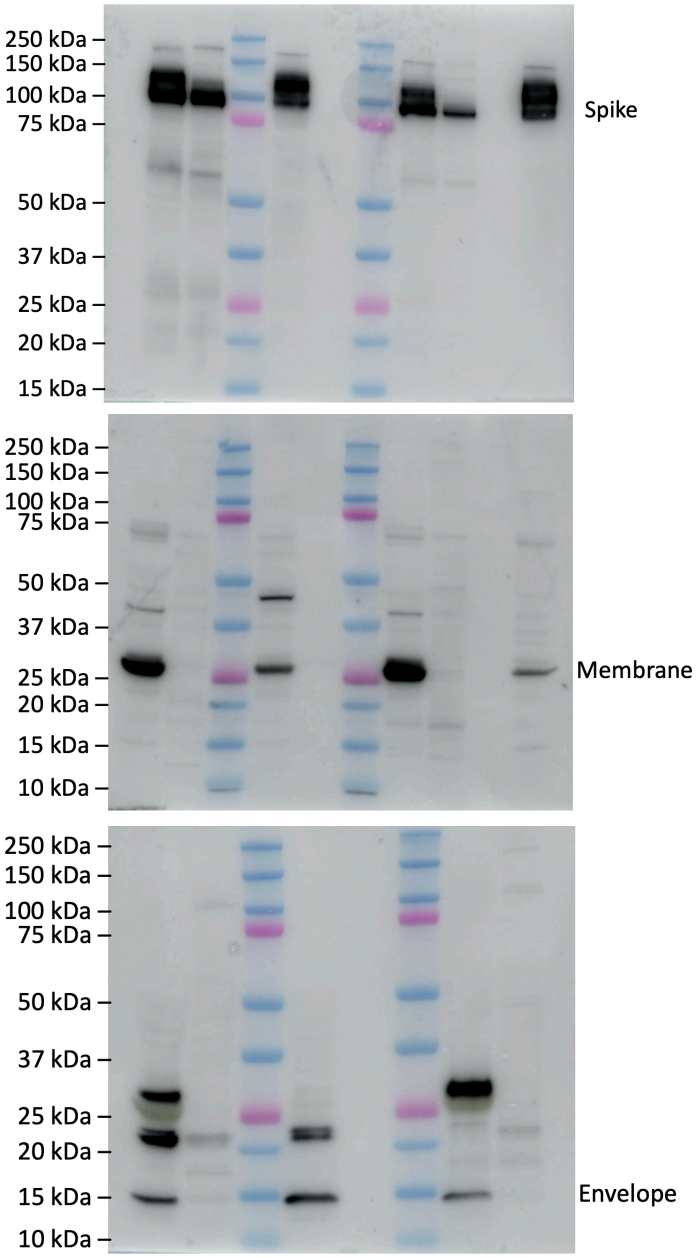

Supplementary Figure 5. Un-cropped and unprocessed images used to generate Fig. 2c, d & e showing S, M and E proteins in purified ß-SARS-CoV-2 VLPs.

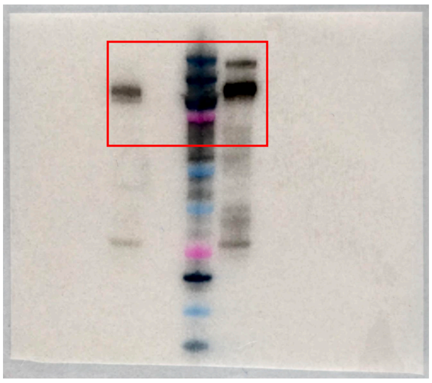

Spike

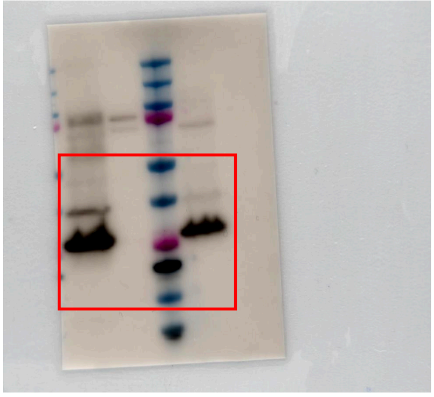

Membrane

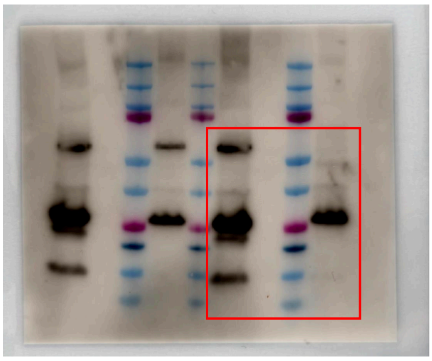

Envelope

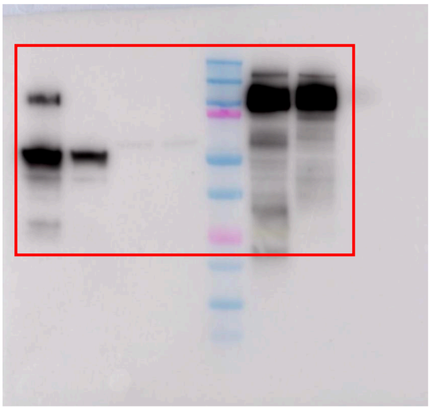

Spike

Supplementary Figure 6. Un-cropped and unprocessed images used to generate Fig. 3c and d showing S, M and E proteins in purified ß-SARS-CoV-2 VLPs (Fig 3C) and immunoprecipitation of ß-SARS-CoV-2 VLPs (Figure 3D).

Red boxes indicate the regions shown in Figure 3C and D. Other bands are not relevant to this paper.

	Primer sequences
ß-S forward	$^{5'}$CGGGTACCGCCACCATGTTCGTGTTTCTG$^{3'}$
ß-S reverse	$^{5'}$CGGCGGCCGCTCATCAGGTGTAGTGCAGTTTCAC$^{3'}$
Wuh $E_{SPP}M$ forward	$^{5'}$TATTAGGTACCGCCACCATGTACAGCTTTGTGTCCGAGGAAACCGGC$^{3'}$
Wuh $E_{SPP}M$ reverse	$^{5'}$TATTTGCGGCCGCTCATCACTGCACC$^{3'}$

Suppl Table 1. Primer sequences for producing recombinant adenoviruses
